# Expanding Clinical Phenotype and Novel Insights into the Pathogenesis of ICOS Deficiency

**DOI:** 10.1007/s10875-019-00735-z

**Published:** 2019-12-20

**Authors:** Hassan Abolhassani, Yasser M. El-Sherbiny, Gururaj Arumugakani, Clive Carter, Stephen Richards, Dylan Lawless, Philip Wood, Matthew Buckland, Marzieh Heydarzadeh, Asghar Aghamohammadi, Sophie Hambleton, Lennart Hammarström, Siobhan O Burns, Rainer Doffinger, Sinisa Savic

**Affiliations:** 1 Division of Clinical Immunology, Department of Laboratory Medicine,, Karolinska Institutet at Karolinska University Hospital Huddinge, Stockholm, Sweden; 2 Research Center for Immunodeficiencies, Pediatrics Center of Excellence, Children’s Medical Center, Tehran University of Medical Sciences, Tehran, Iran; 3 NIH Research-Leeds Biomedical Research Centre and Leeds Institute of Rheumatic and Musculoskeletal Medicine (LIRMM), Wellcome Trust Brenner Building, St. James’s University Hospital, Beckett Street, Leeds, UK; 4 Clinical Pathology Department, Faculty of Medicine, Mansoura University, Mansoura, Egypt; 5 Department of Biosciences, School of Science and Technology, Nottingham Trent University, Nottingham, UK; 6 Department of Clinical Immunology and Allergy, St. James’s University Hospital, Leeds, UK; 7 Haematological Malignancy Diagnostic Service, Leeds Teaching Hospitals NHS Trust, Leeds, UK; 8 Leeds Institute of Biomedical and Clinical Sciences, University of Leeds, Wellcome Trust Brenner Building, St James’s University Hospital, Beckett Street, Leeds, UK; 9 Institute of Immunity and Transplantation, Division of Infection & Immunity, School of Life and Medical Sciences, University College London, Royal Free Hospital, London, UK; 10 Department of Pediatrics and Neonatology, School of Medicine, Kashan University of Medical Sciences, Kashan, Iran; 11 Primary Immunodeficiency Group, Institute of Cellular Medicine, Medical School, Newcastle University, Newcastle upon Tyne, UK; 12 Department of Clinical Biochemistry and Immunology, Addenbrooke’s Hospital, Cambridge, CB2 2QQ UK

**Keywords:** Primary immunodeficiency, granulomatous inflammation, chronic diarrhea, antibody deficiency, ICOS deficiency, ustekinumab

## Abstract

**Background:**

Inducible T cell co-stimulator (ICOS) deficiency has been categorized as a combined immunodeficiency often complicated by enteropathies, autoimmunity, lymphoproliferation, and malignancy. We report seven new patients and four novel *ICOS* mutations resulting in a common variable immunodeficiency (CVID)–like phenotype and show that dysregulated IL-12 release, reduced cytotoxic T lymphocyte–associated protein 4 (CTLA4) expression, and skewing towards a Th1-dominant phenotype are all associated with inflammatory complications in this condition.

**Methods:**

A combination of whole exome and Sanger sequencing was used to identify novel mutations. Standard clinical and immunological evaluation was performed. FACS and ELISA-based assays were used to study cytokine responses and ICOS/ICOSL/CTLA4 expression following stimulation of whole blood and PBMCs with multiple TLR ligands, anti-CD3, and PHA.

**Results:**

Four novel ICOS mutations included homozygous c.323_332del, homozygous c.451C>G, and compound heterozygous c.58+1G>A/c.356T>C. The predominant clinical phenotype was that of antibody deficiency associated with inflammatory complications in 4/7 patients. Six out of seven patients were treated with immunoglobulin replacement and one patient died from salmonella sepsis. All patients who were tested showed reduced IL-10 and IL-17 cytokine responses, normal IL-1β, IL6, and TNF release following LPS stimulation and highly elevated IL-12 production in response to combined LPS/IFNγ stimulation. This was associated with skewing of CD4^+^ T cells towards Th1 phenotype and increased expression of ICOSL on monocytes. Lastly, reduced CTLA4 expression was found in 2 patients. One patient treated with ustekinumab for pancytopenia due to granulomatous bone marrow infiltration failed to respond to this targeted therapy.

**Conclusions:**

ICOS deficiency is associated with defective T cell activation, with simultaneously enhanced stimulation of monocytes. The latter is likely to result from a lack of ICOS/ICOSL interaction which might be necessary to provide negative feedback which limits monocytes activation.

**Electronic supplementary material:**

The online version of this article (10.1007/s10875-019-00735-z) contains supplementary material, which is available to authorized users.

## Introduction

Inducible T cell co-stimulator (ICOS) is a member of the CD28/CTLA4 family, which plays an important role in regulating T cell–mediated immune responses as a secondary co-stimulatory signal delivered in concert with T cell receptor stimulation [1, 2]. Unlike CD28, ICOS is not constitutively expressed, but it is exclusively upregulated on activated T cells. Although these two co-stimulatory molecules share several intracellular signaling pathways [3, 4], their functions are only partially overlapping, including cell survival, proliferation, and differentiation. Several in vitro and animal studies have shown important roles of ICOS in the induction and regulation of Th1, Th2, and Th17 immunity [5, 6]. Other effects of ICOS on the immune system are further determined by the cellular location of its unique ligand (ICOSL), which has a broad tissue expression including immune cells such as monocytes, dendritic cells, and B cells [7–9]. In the latter, ICOS/ICOSL interaction and the ensuing cytokine production contributes to immunoglobulin (Ig) production and long-lived switched memory/plasma cell development within germinal centers [10].

ICOS deficiency has been categorized as a combined immunodeficiency [11] and the clinical features include hypogammaglobulinemia, vulnerability to infections, enteropathies, autoimmunity, lymphoproliferation, and malignancy [12–17]. Although *ICOS* was the first described gene associated with a common variable immunodeficiency (CVID)–like phenotype, to date, only four different mutations have been identified in a total of 15 patients [18]. Therefore, ICOS deficiency accounts for less than 1% of approximately 2600 CVID-like patients with known gene defects (including *TNFRSF13B* (80%), *TNFRSF13C* (4%), *LRBA* (2.5%), and *PIK3CD* (2.5%)) [12–17, 19].

Here we report 7 new patients presenting with four novel *ICOS* mutations resulting in a CVID phenotype, including the first 2 cases ever reported of ICOS deficiency due to a missense mutation located within the helical domain of the protein, and another 2 patients with a compound heterozygous mutations, one of which is located at a splice site. We compared the clinical and immunological features of these patients with previously published cases. Furthermore, we show that inflammatory complications associated with ICOS deficiency might be due to a skewed Th1 response, resulting from excessive IL-12 production caused by the failure to downregulate ICOSL expression on antigen presenting cells, including dendritic cells and monocytes. Finally, we report on treatment outcome following a trial of the IL-12/23 blocker ustekinumab in a patient with granulomatous complications.

## Materials and Methods

### Patients and Clinical Evaluation

Evaluation of medical records was carried out after obtaining written informed consent, performed in accordance with the guidelines of the ethics committees or the Institutional Review Boards of participating institutes at St. James’s University Hospital, University College London (Royal Free Hospital), and the Tehran University of Medical Sciences. All patients were diagnosed as antibody deficient based on the updated diagnostic criteria of ESID (the European Society for Immunodeficiencies, http://esid.org/WorkingParties/Registry/Diagnosis-criteria) [20] and AAAAI (The American Academy of Allergy, Asthma and Immunology) practice parameter for the diagnosis and management of primary immunodeficiency [21]. After confirmation of diagnosis, patients were classified according to the International Union of Immunological Societies (IUIS) and Primary Immunodeficiency Diseases Committee updated classification [11]. An evaluation sheet was used to summarize the demographic information of patients including gender, ethnicity, place and date of birth, medical history including the date of diagnosis and record of other diseases, clinical manifestations, relevant laboratory tests, and family history.

### Routine Immunological Assays

Complete blood count, lymphocyte subpopulations, serum Ig levels, and specific antibody responses were measured as previously described [22–25].

### Genetic Evaluation and In Silico Analysis

Whole exome sequencing (WES) was performed according to established protocols [19, 22, 26]. The pathogenicity of all disease attributable gene variants was re-evaluated using the updated guideline for interpretation of molecular sequencing by the American College of Medical Genetics and Genomics (ACMG), considering allele frequency in the population databases, computational data, immunological/functional data, familial segregation and parental data, and clinical phenotyping [27]. Existing databases including the 1000 Genome; Exome Aggregation Consortium; National Heart, Lung, and Blood Institute exome variant server; and Greater Middle East Variome were used for filtering based on the population frequency for any given variant for all families. ICOS has a gene damage index of 1.51 and a mutation significance cutoff of 5.374 [28]. The combined annotation-dependent depletion scores [29] for the ICOS variants identified in the patients were calculated and compared with the gene-specific mutation significance cutoff.

### Confirmatory Sequencing

Genomic DNA was used in PCR employing exon-specific primers (Supplementary Table E1). The PCR products were purified using the QIAquick gel extraction kit (Qiagen) and subsequently sequenced at Macrogen Inc. The sequences were analyzed using the Lasergene software package (DNAStar).

### Th1/Th2 Phenotyping, ICOS, ICOSL, and CTLA4 Expression

Peripheral blood mononuclear cells (PBMCs) from patients and healthy donors were separated using a density gradient method (Lymphoprep™, Alere Technologies, Norway) from ethylenediaminetetraacetic acid (EDTA) anticoagulated peripheral blood. Red cell lysis with ammonium chloride was performed for Th1/Th2 phenotyping. The isolated cells were washed twice by Dulbecco’s phosphate-buffered saline (DPBS) then labeled with a panel of monoclonal antibodies for immunophenotyping. Analysis with multi-parameter 15 colors flow cytometry panel was performed. The data was acquired on the Becton Dickinson (BD) LSR II flow cytometer or BD FACSLyric instruments, and the data was analyzed using the BD FACSDiva software version 8.0. (for gating strategy please see Supplementary Fig. E1).

For PBMCs stimulation, cells were incubated in RPMI 1640 Medium, GlutaMAX™ Supplement 10% fetal calf serum (FCS) all from (Thermofisher, USA) at density of 1 × 10^6^ cells/ml, and were stimulated using 5 μg/ml for phytohemagglutinin (PHA) 10 μg/ml, lipopolysaccharide (LPS) 10 ng/ml both from (Sigma-Aldrich) and/or crosslinking beads of Anti-Biotin MACSiBead particles and biotinylated antibodies against human CD2, CD3, and CD28, were used as recommended by manufacturer 1 cell/1 bead (Miltenyi Biotec, Germany). Cells were analyzed using a multi-parameter flow and for ICOSL surface expression based on the gating strategy.

Cell surface and intracellular levels of CTLA4 were measured on T effector cells (CD3^+^ CD4^+^ CD25^+^) and regulatory (CD3^+^ CD4+ CD25^hi^ Foxp3^+^) T cells using antibody staining and flow cytometry. Peripheral blood mononuclear cells were isolated by centrifugation (400*g* for 20 min) over Lymphoprep (Cedar lane) and washed in TC199 tissue culture medium. Cells were stained both prior to culture (fresh), and following culture in 96-well plates in triplicate in the presence of PHA (Sigma, 2.5 μg/ml) for 48 h at 37 °C. For cell surface expression of CTLA4, cells were stained with CD3, CD4, CD25, and CTLA4, prior to fixation, permeabilization, and staining with FOXp3 antibody. For intracellular staining of CTLA4, surface stained (CD3^+^ CD4^+^ CD25^+^) PBMC were fixed and permeabilized using BD Pharmingen human FoxP3 buffer set (Cat No. 560098) according to manufacturer’s instructions. Cells were subsequently stained with Foxp3/CTLA4 antibodies for 30 min at room temperature prior to the final washing steps and suspension in PBS/formaldehyde. Data acquisition was performed using a FACS Canto II flow cytometer, using the FACSDiva software with approximately 10^4^ CD4^+^ T cells acquired. For all details regarding specific antibodies used for flow cytometry, please see Supplementary methods.

### Functional Analysis of Cytokine Production

Whole blood of patient and controls was diluted 1:5 in RPMI into 96-well F plates (Corning) and activated by single stimulation with phytohemagglutinin (PHA, 10 μg/ml; Sigma-Aldrich) or LPS (1 μg/ml, List Biochemicals) alone or by co-stimulation with IFN-γ (2 × 10^4^ IU/ml, Imukin, Boehringer Ingelheim), and supernatants were taken after 24 h incubation at 37 °C/5%CO_2_. Cytokines were measured by multiplexed bead array (IFN-γ, TNF-α, IL-12, IL-10, IL-6, IL-17A, IL-1β, R+D Systems Fluorokinemap) on a Luminex analyzer (Bio-Plex, Bio-Rad, UK).

### Statistical Analysis

Statistical analyses were performed using the SPSS software package, version 22 (SPSS Inc., Chicago, IL, USA). Two-way ANOVA statistical test was used for comparison of cytokine production between patients and controls (*p* < 0.05). Kaplan-Meier curve and 95% confidence interval survival analysis were utilized for prognosis evaluation of the patients.

## Results

### Genetic Diagnosis, Mutation Analysis, and Protein Expression

**Fig. 1:**
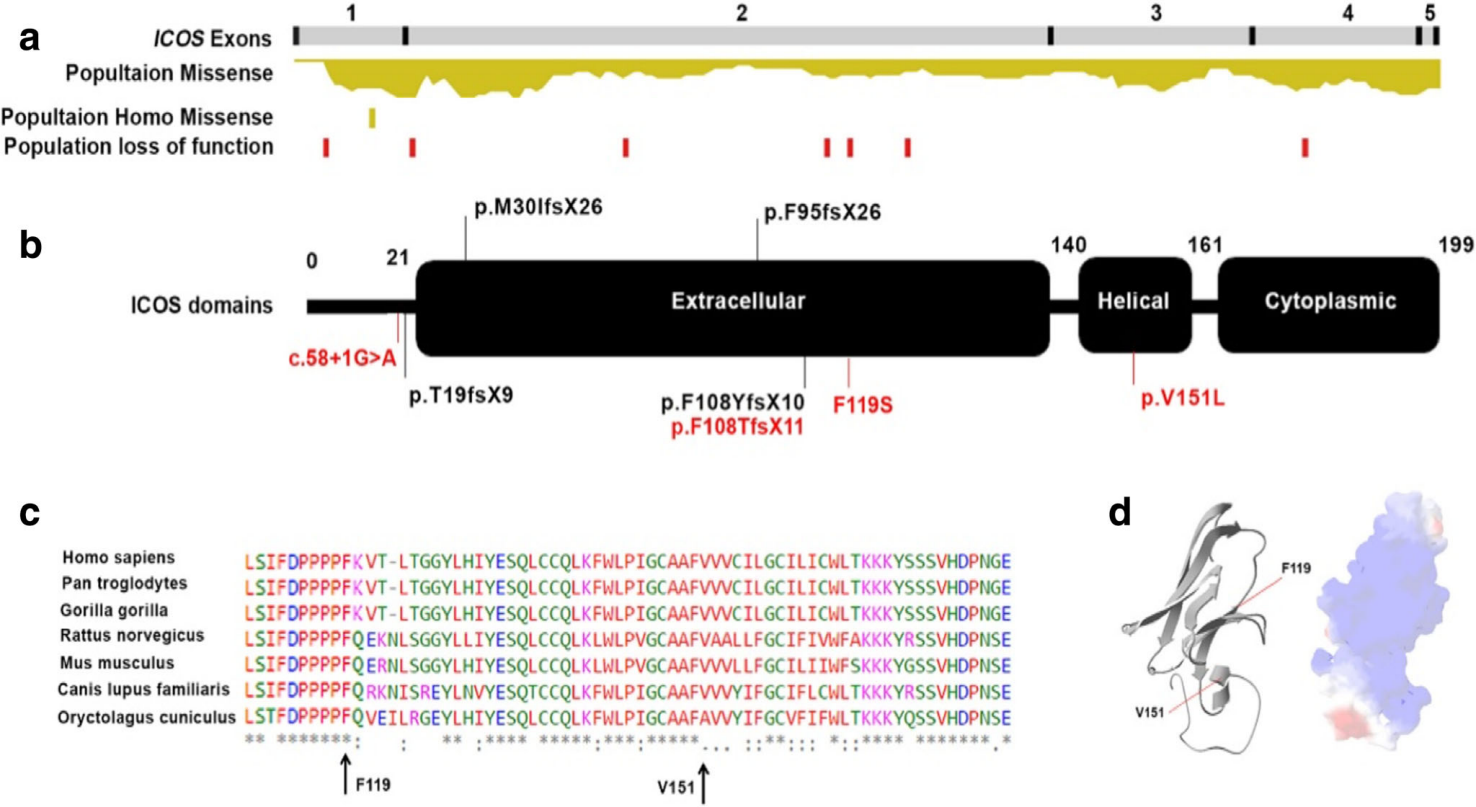
ICOS variants diagnosed in combined immunodeficiency. **a** Schematic view of the ICOS exons and domains representing the rate of missense and loss of function mutation among a normal population (extracted from genomAD), variants diagnosed in patients with ICOS deficiency. **b** Black and red mutations marks refer to previously reported and newly identified mutations, respectively. **c** Multiple sequence alignments representing conservation within positions of newly observed missense mutations (p.F119S and p.V151L) in the ICOS protein. **d** 3D structure of ICOS protein representing the location of the missense mutations in helical domain of the protein

We combined the information from previously published cases (P1–P15) [12–17] and our new patients (P16–P22) to provide an up to date clinical overview of ICOS deficiency. Since all probands were diagnosed clinically with hypogammaglobulinemia, the known genes associated with antibody deficiency were excluded using next-generation sequencing (Supplementary Table E2) [19]. Three patients (P16, P17, and P18), all of the Pakistani background but not from the same kindred, were found to have a novel homozygous deletion c.323_332del (p.F108TfsX11) (Supplementary Fig. E2). To our knowledge, P19 and P20 are the first ICOS deficiencies caused by compound heterozygous mutations (p.F119S and c.58+1G>A), one of which is a splice site mutation (Supplementary Fig. E2). P21 and P22 were homozygous for a novel missense mutation (p.V151L, Fig. 1, Supplementary Fig. E2, and Fig. E3 within the helical domain. In silico analysis of the identified missense mutations (p.F119S and p.V151L) showed that both are located in highly conserved residues and both damage the formation of protein and nucleotide binding regions surrounding the helical domain, suggesting the production of a misfolded protein (Fig. 1 and supplementary Fig. E3).

We studied ICOS protein expression in P16, P17, P19, and P20. As expected, neither P16 nor P17 (with homozygous deletion mutations) had any expression of ICOS on CD4^+^ T cells following stimulations with anti-CD3 and PHA (Supplementary Fig. E4). There was also no ICOS expression in P19 or P20 despite the fact that the point mutation (p.F119S) was not predicted to lead to a complete loss of protein expression. Interestingly, F119S mutant was described previously as part of in vitro study to map out ligand binding sites for ICOS. This mutant can be expressed in vitro; however, this was in a complex with CH2-CH3 portion of human IgG1. The mutated protein, although expressed still failed to bind to B7-H2 [30].

### Clinical and Immunological Overview of ICOS Deficiency

The summary of clinical and immunological features of the 7 new cases is shown in Table 1. Detailed clinical reports are provided in Supplementary results. All patients had a prior diagnosis of antibody deficiency, with 6/7 labeled as CVID.

**Table 1:** Main clinical and immunological features of ICOS deficiency

Patients	Total *n* (%)	P1	P2	P3	P4	P5	P6	P7	P8	P9	P10	P11	P12	P13	P14	P15	P16	P17	P18	P19	P20	P21	P22
Respiratory features	17 (77.2)	+	+	+	+	+	+	+			+		+		+		+	+	+	+	+	+	+
Bronchiectasis	3 (13.6)			+				+													+		
Severe viral infections	11 (50)	+	+			+	+	+		+	+		+		+		+				+		
Opportunistic infections	5 (22.7)		+			+							+		+			+					
Autoimmunity	7 (31.8)						+	+		+	+	+						+		+	+		
Enteropathies	13 (59.0)		+		+			+		+	+		+	+	+	+				+	+	+	
Lymphoproliferation	9 (40.9)	+	+		+	+	+			+					+		+	+					
Malignancy	2 (9.0)	+					+																
Deceased	4 (18.1)	+			+*										+			+					
Hypogammaglobulinemia	22 (100)	+	+	+	+	+	+	+	+	+	+	+	+	+^**^	+	+	+	+	+	+	+	+	+
Normal IgG3	5 (29.4)				+	+		+				+		+			NR	NR	NR	NR	NR		+
Low IgM	12 (54.5)	+	+	+	+	+		+							+	+		+	+		+	+	+
Normal IgM	5 (22.7)			+		+					+	+	+										
Normal IgA	5 (22.7)											+		+	+	+					+		
Specific antibody deficiency	16 (88.8)	+	+	+	+	NR	NR	NR	+	+	+	+	NR	+	+	+		+	+	+	+	+	
Anemia	6 (28.5)				+		+	+		+	+			NR			+						
Thrombocytopenia	7 (33.3)	+			+	+	+				+		+	NR			+						
Leukopenia	4 (19.0)					+	+						+	NR			+						
Neutropenia	2 (9.5)					+	+							NR			+						
Lymphopenia	5 (23.8)		+	+	+	+	+							NR			+^#^						
T cell lymphopenia	5 (23.8)				+	+	+						+	NR									
CD4^+^ T cell lymphopenia	7 (33.3)		+		+	+	+	+					+	NR									
CD8^+^ T cell lymphopenia	8 (38.0)			+	+	+	+				+	+	+	NR									
B cell lymphopenia	12 (60.0)	+	+	+	+	+	+	+	+	+	+	+		NR				+					
NK lymphopenia	8 (38.0)	+	+		+	+	+					+	+	NR		+	+						
Increased naïve B cells	15 (93.7)	+	+	+	+	+	+	+	+	+	NR	NR	+	NR	+	+	+	NR	NR	+		+	+
Reduced non-SM B cells	16 (88.8)	+	+	+	+	+		+	+	+	+	+	+	NR	+	+	+	NR	NR	+		+	+
Reduced SM B cells	17 (100)	+	+	+	+	+	+	+	+	+	+	+	+	NR	+	+	+	NR	NR	NR	+	+	+
Increased naïve CD4^+^ T cells	5 (33.3)			+					+	+	+	+	NR	NR			NR	NR	NR	NR	NR		
Reduced effector CD4^+^ T cells	5 (38.4)			+					+	+	+	+	NR	NR	NR	NR	NR	NR	NR	NR	NR		
Reduced follicular CD4^+^ T cells	11 (100)	+	+	+	+	+	+	+	+	+	NR	NR	NR	+	NR	+	NR	NR	NR	NR	NR	NR	NR

*Due to non-immunodeficiency related condition

**Mild transient hypogammaglobulinemia and specific antibody deficiency

^#^Developed later not present at the diagnosis

*NR*, not reported; *SM*, switched memory

**Fig. 2:**
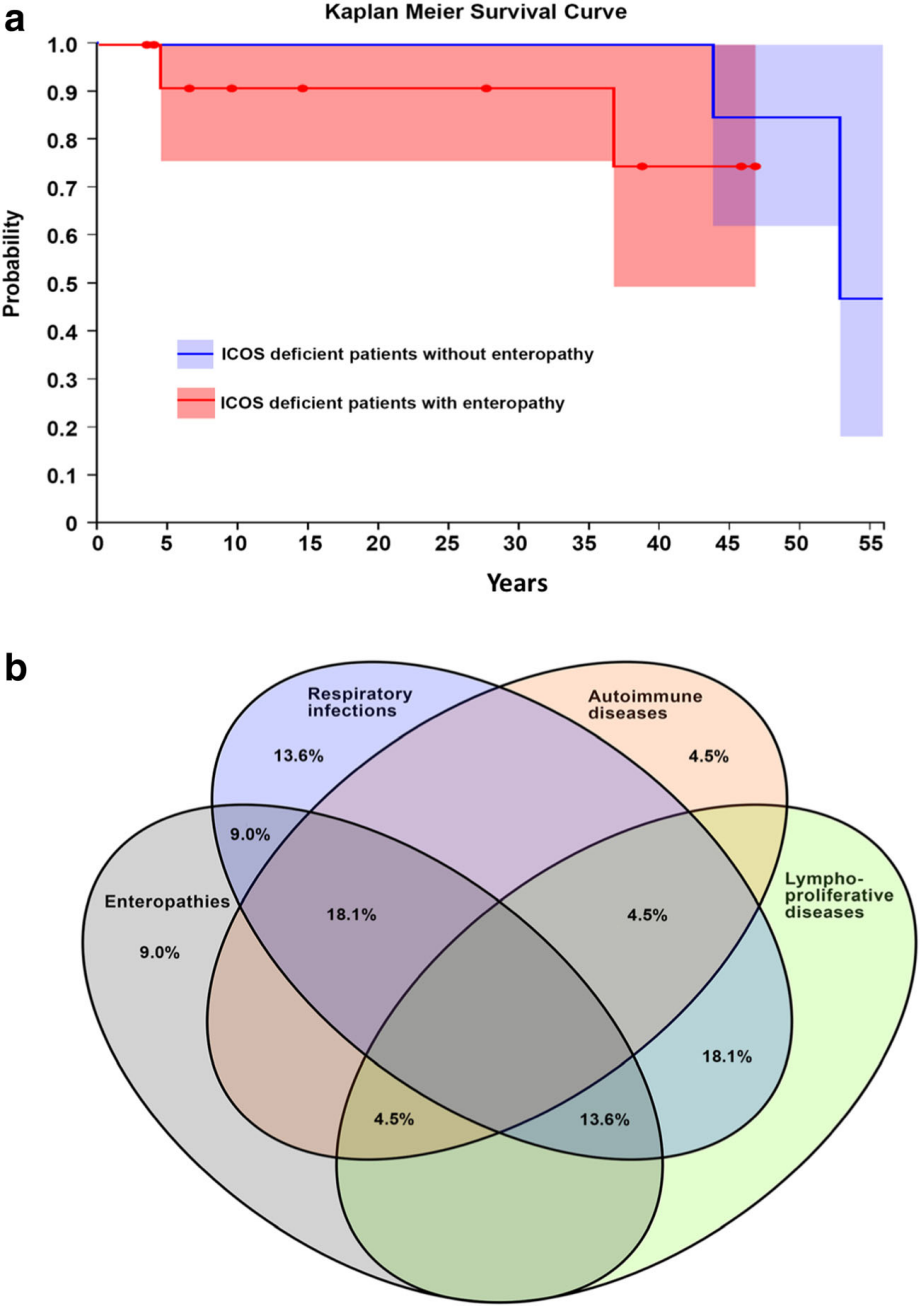
**a** Kaplan-Meier survival rate for ICOS deficiency. **b** Clinical features of ICOS deficiency

The clinical features observed in all patients with ICOS deficiency (Table 1, Supplementary Tables E3–7, and Fig. 2) are heterogeneous with the age of presentation ranging from early infancy to 39 years. The majority (70%, 14/20) presented at childhood or before 13 years of age, but due to the piecemeal progression and mild phenotype, most of the patients were diagnosed in adulthood (68.1%, 15/22). The disease phenotype can broadly be divided into an infectious phenotype (77.2%), an enteropathy phenotype (54.5%), a lymphoproliferative phenotype (40.9%), and an autoimmunity phenotype (31.8%, Fig. 2b).

The infections include encapsulated bacterial infections due to hypogammaglobulinemia mainly in the respiratory system, but also viral infections (particularly by the *Herpesviridae* family) and opportunistic fungal and parasitic infections, suggesting some degree of combined immunodeficiency (Supplementary Tables E4–5). *Pneumocystis jirovecii* pneumonia was also described in one report [17]. A wide range of autoimmune diseases has been documented, including autoimmune cytopenia, autoimmune interstitial pneumonitis, inflammatory bowel disease, psoriasis, and rheumatoid arthritis.

Enteropathy usually manifested as chronic diarrhea without significant growth retardation. The lymphoproliferative spectrum consisted of lymphadenopathy, hepatosplenomegaly, granulomatous lesions, and nodular lymphoid hyperplasia. Carcinoma of the vulva associated with human papillomavirus (HPV) infection was also reported in one patient (P1) and squamous cell carcinoma in one patient (P6). It appears that chronic diarrhea dominated in patients presenting before adolescence and was associated with viral infections (P12–P15 and P19–21), while respiratory infections and cutaneous lesions were more prominent in older patients (P1–P10). However, single episodes of pneumonia in childhood did occur in some patients prior to developing recurrent respiratory and other complications and a subsequent diagnosis of CVID in adulthood (P1–P9). This temporal gap in presentation, where symptoms occur in early life followed by a dormant period, occurred in one third of the patients (5 out of 15, Table 1) reported to date.

P16 had further unusual complications, not previously described in ICOS deficiency. He developed well-documented delayed type hypersensitivity reactions to several different antibiotic classes (Supplemental results, Fig. E5**a**). Interestingly, lymphocyte transformation assays to all implicated antibiotics were negative (Supplementary Fig. E5b).

### Immunological Features

Human ICOS deficiency abrogates the germinal center reaction and provides a monogenic cause of CVID immunological phenotype in most of the patients. However, hyper IgM phenotype has also been reported in two patients (P11 and P12, 9.0%). Antibody responses to vaccination using different antigens result in non-protective IgG production (especially against peptide antigens); however, one patient (P22) showed intact specific antibody production.

Peripheral B cell counts may be reduced, particularly in adulthood and accompanied by a reduction in naïve B lymphocyte counts (8 out of 19, 42.1%); whereas, almost all ICOS-deficient patients present an increased level of naïve B lymphocytes (93.7%) at the time of diagnosis. ICOS-deficient patients show reduced numbers of both CD27^+^ switched memory B cells (100%) and non-switched memory B cells (88.8%). The ICOS-deficient patients show an almost normal distribution of T cell subpopulations (except for a decreased number of CXCR5^+^CD4^+^ T helper cells in the all studied patients and an inverted CD4^+^/CD8^+^ ratio in 33.3%). Natural killer (NK) lymphopenia was also recorded in 38% of studied patients, suggesting a degree of innate immune defects in the ICOS deficiency. Table 1 and Supplementary Tables E6–7 depict all immunological manifestation of the 22 ICOS-deficient patients reported to date.

### Functional Analysis of Cytokine Responses

**Fig. 3:**
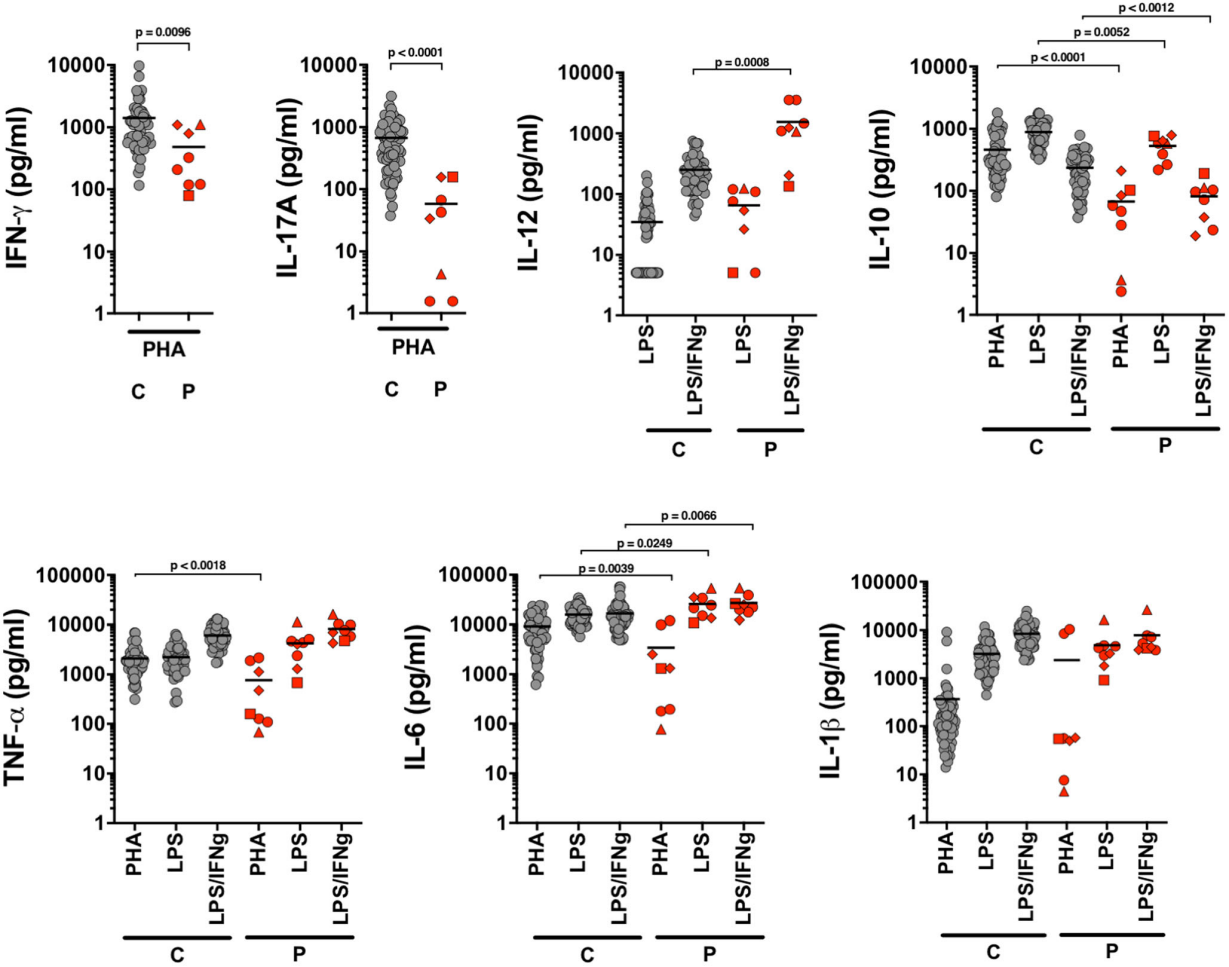
Cytokine production after whole blood stimulation: healthy controls (*C* gray circles, *n* = 59) and patients (P16, red circles; P17, red triangles; P18, red squares; P20, red diamonds). Statistical analysis was done with two-tailed Mann-Whitney tests using the GraphPad Prism software V8.02

Poor IL-10 and IL-17 production were previously demonstrated in ICOS deficiency [14]. Furthermore, one report showed inadequate production of pro-inflammatory cytokines, IL-1, IL-6, and TNF [15]. We tested the cytokine production using whole blood from P16, P17, P19, and P20. We replicated significantly poorer IL-10 and IL-17 responses to polyclonal T cell stimulation with PHA in all patients when compared with HC. However, we found that production of IL-1ß and TNF was comparable with HC in response to LPS and IFNγ/LPS. Interestingly, ICOS-deficient patients showed significantly higher IL-6 production following LPS or combined IFNγ/LPS stimulation of the whole blood. Similarly, significantly elevated production of IL-12 was seen in almost all patients compared with HC after combined IFNγ/LPS stimulation (Fig. 3).

### ICOSL Expression

**Fig. 4:**
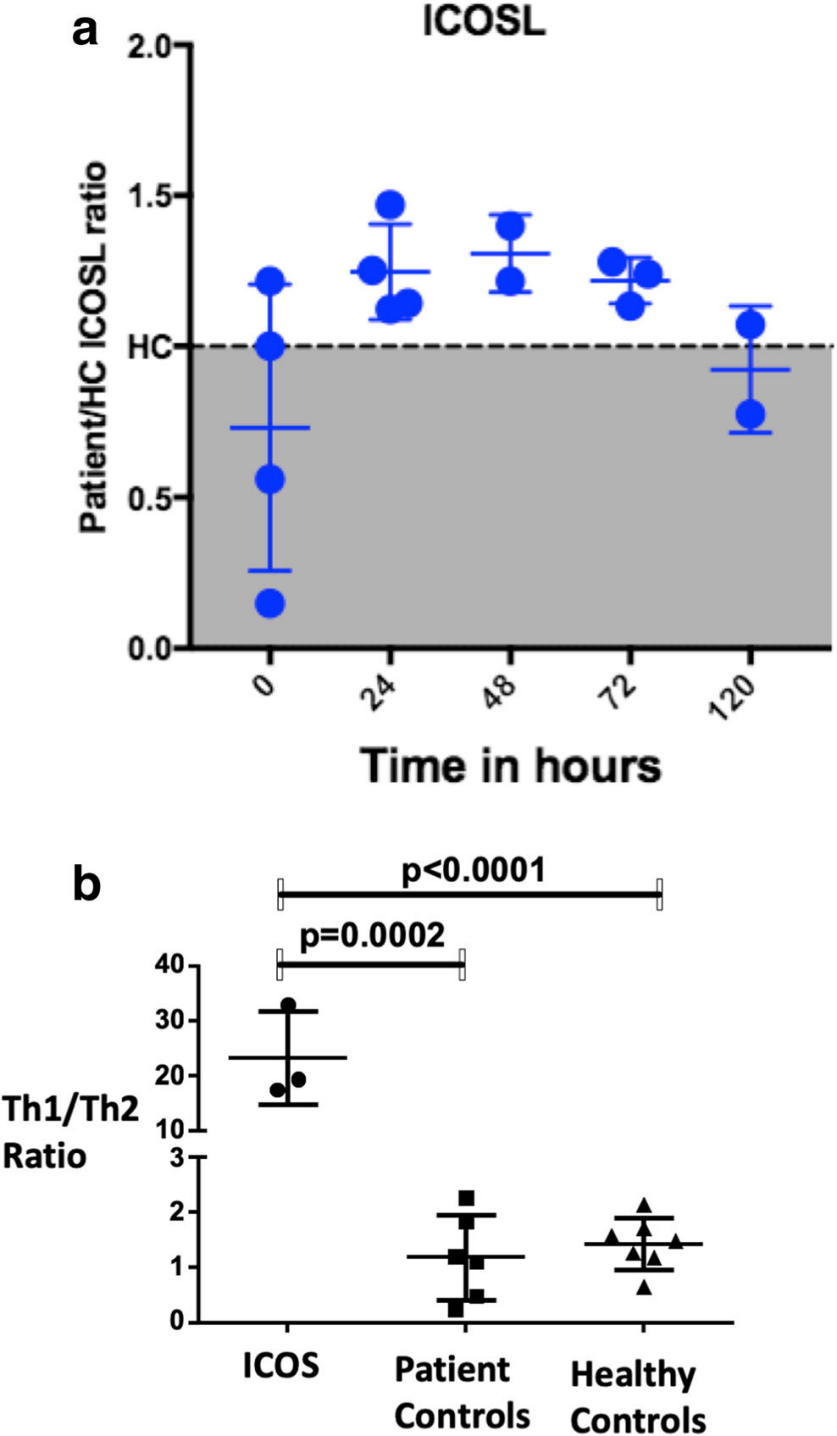
ICOSL expression on monocytes and Th1 and Th2 ratios. **a**. Time line changes (0–120 h) ICOSL level fold change on monocytes in patients (*n* = 4) determined by flow cytometry compared with health controls (*n* = 3) following LPS stimulation. **b** Mean with SD of the ratio of Th1/Th2 cells within the memory T cell population (CD45RA Neg CD4 T cells) of ICOS deficiency (*n* = 3), ICOS sufficient primary antibody deficiency (*n* = 6), and healthy controls (*n* = 7). Th1 cells were gated as CD183+CD196− helper memory T cells and Th2 cells were gated as CD183−CD196− helper memory T cells. Unpaired *t* test and *p* values provided. The detailed gating strategy is provided in Fig. E4.

Monocytes and dendritic cells (DCs) are typically the main producers of IL-12. We wondered whether lack of ICOS expression on T cells, and therefore lack of engagement with ICOSL expressed by the activated monocytes and DC, might be causing a persistent activation of the latter, and increased production of IL-12. We therefore investigated ICOSL expression on monocytes from the patients and HC following stimulation with LPS over several days. We found significantly increased expression of ICOSL on patient monocytes at 24 h, 48 h, and 72 h time points, with the levels returning to those comparable with HC at day 4 (120 h) (Fig. 4a). This finding is consistent with a murine model which shows that both ICOS and ICOSL depend on their mutual interaction to regulate their surface expression [31].

### TH1/TH2 Profile

As IL-12 has an important role in driving Th1 differentiation, we performed detailed T cell phenotyping of P16 and P17. In both cases, there was a significant skewing of the T cell phenotype towards Th1 (Fig. 4b). The ratio of Th1/Th2 cells was significantly elevated compared with ICOS sufficient primary antibody deficiency patients (*p* = 0.0002) and healthy controls (*p* < 0.0001). The predominance of Th1 phenotype in ICOS deficiency was seen in both T follicular helper T cells and non-follicular T helper cells.

### CTLA4 Expression

**Fig. 5:**
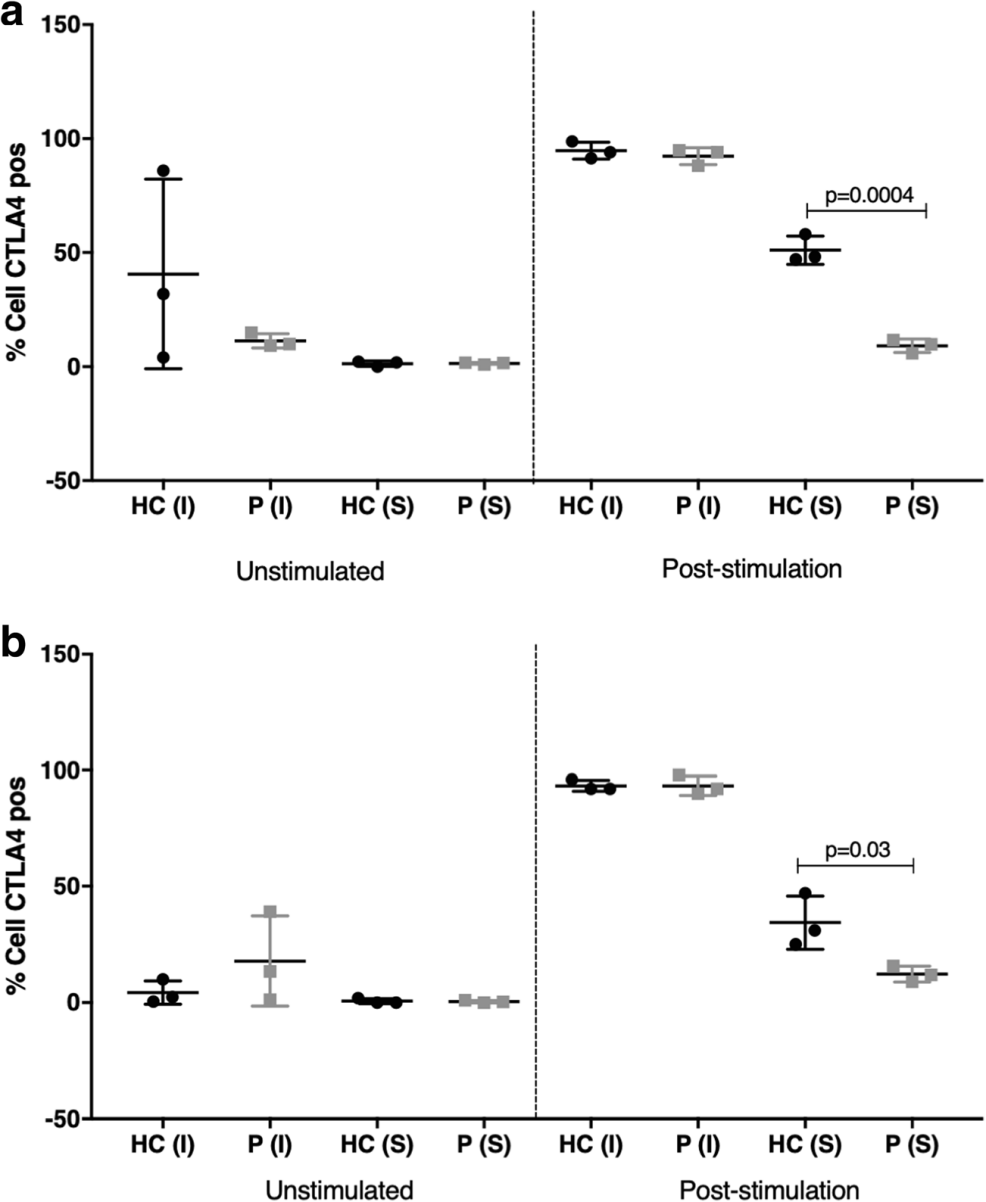
CTLA expression on **a** Tregs and **b** CD4+ T effector cells. PBMC were isolated from healthy control (HC) and patients (P16 and P17). Cells were stained both prior to culture (fresh), and following culturing in 96-well plates in triplicate in the presence of PHA (Sigma, 2.5 μg/ml) for 48 h at 37 °C. Surface and intracellular levels of CTLA4 were assessed on both Tregs and effector T cell populations. The figure represents the average of 3 separate experiments. *S*, surface expression; *I*, intracellular expression. Statistical analysis was done with unpaired *t* test and *p* values provided tests using the GraphPad Prism software V8.02

Lastly, we examined the expression of CTLA4 on all CD4^+^ T cells and specifically on the regulatory T cells from P16 and P17. Since CTLA4 expression on T cells is not constitutive but dependent on their activation, we postulated that CTLA4 expression in ICOS deficiency might be impaired due to inadequate T cell activation. We stimulated T cells from P16 and P17 with PHA (Sigma, 2.5 μg/ml) for 48 h at 37 °C and examined the intracellular and extracellular expression of CTLA4 at the baseline and following stimulation with PHA. We found that in both patients, CD4 effector T cells (CD3^+^ CD4^+^ CD25^+^) failed to upregulate CTLA4. A similar finding was observed in regulatory T cells (CD3^+^ CD4^+^ CD25^hi^ FOXp3^+^, CD127^−^), which interestingly showed comparable intracellular expression of CTLA4 with HC, but the expression of CTLA4 on the cell surface was significantly reduced (Fig. 5).

### Treatment Outcomes

Regarding the newly identified cohort, 6/7 patients received intravenous Ig (IVIg), one patient was only on prophylactic antibiotic treatment (Table 1), and one (P17) died at the age 53 from overwhelming salmonella sepsis. A previous report describes premature death in three patients (malignancy in P1, capillary leak syndrome, and toxic epidermal necrolysis after hematopoietic stem cell transplantation (HSCT) in P14, and due to an immunodeficiency unrelated event in P4). However, transplantation has been successfully used in two ICOS patients (P6 and P12). Although the Kaplan-Meier 55-year survival rate of ICOS deficiency is estimated to be 81% (CI 0.72–1.0) the rate of mortality seems higher in patients with enteropathy phenotype (Fig. 2a); however, this is not statistically significant. Moreover, death in infancy was also reported in siblings of patients (in P10, P11, and P17).

Interestingly, one female patient was reported to be asymptomatic despite being homozygous for a 1815 base pair deletion (P8), but Ig substitutions have been administrated on this patient [18]. P18 in our cohort, showing very mild presentation and only respiratory infections was also female. Of note patients diagnosed to date with symptomatic ICOS deficiency are mainly males (14/22, 63.6%). This discrepancy in the disease severity was also seen in two other sibling pairs in the new cohort. Patient P19 has a relatively mild and predominantly infectious phenotype, unlike his sister (P20) who had severe and multiple inflammatory/autoimmune complications. Disease severity varied in a similar fashion between P21 and P22, despite them sharing the same novel homozygous missense mutation. This suggests that a wide range of clinical presentations is probably the result of other modifying factors.

Patient 16, who had multiple inflammatory and autoimmune complications, received ustekinumab (Stalara) (260 mg IV followed by 90 mg IV after 8 weeks followed by 90 mg IV every 12 weeks) having previously failed high dose IVIg, corticosteroids (Prednisolone 30 mg), and azathioprine to treat multi-linage cytopenias due to granulomatous bone marrow inflammation. As we demonstrated high ex vivo whole blood IL-12 production in this patient, associated with the skewed Th1 profile, we believed that a combined and targeted IL12/IL23 blockade provided by ustekinumab would help to resolve the inflammatory complications without further significant immunosuppressive effect. Unfortunately, despite receiving 6 months of treatment, no significant improvement in the platelet count, neutrophil count, or hemoglobin levels was observed.

## Discussion

The clinical features of ICOS deficiency patients that have been reported worldwide, including the 7 described in this study, suggest that there is no clear genotype-phenotype correlation since patients with the same mutation may exhibit different disease severity and complications or even be asymptomatic; whereas, the immunological features are more consistent. Hypogammaglobulinemia is almost universal, typically associated with abnormal germinal center development and poorly developed memory B cell compartment resulting in a diminished secondary immune response [12, 16, 17]. T cell lymphopenia appears to be rare as we found in our cohort, but there is usually a normal distribution of T cell subpopulations, and T cell proliferative responses are typically preserved. However, it is evident that the lack of ICOS expression on activated T cells decreases IL-10 and IL-17 synthesis and results in a lack of T cell support for B cell maturation and formation of germinal centers. Patients with ICOS deficiency may have either normal [12] or reduced [15] proportions of both CD4^+^ and CD8^+^ memory T cells, and it has been suggested that ICOS is required for the proliferation and survival of memory T cell subsets in humans [15]. In a recent report on memory T cells for two ICOS-deficient patients, the proportion of both central and effector memory cells in helper and cytotoxic T cells were decreased as a consequence of a homozygous p.F95fsX26 mutation (P10–P11) [15].

In this study, we have replicated poor IL-10 and IL-17 cytokine responses. However, we have also shown that the production of pro-inflammatory cytokines is preserved and in some cases enhanced. The previous study which reported diminished IL-1β, IL-6, and TNF production in ICOS-deficient patients used different experimental conditions based on isolated T cells [15]. We performed these investigations using a whole blood assay, which contained monocytes and DCs, and typical producers of pro-inflammatory cytokines. We showed that production of TNF and IL-1β is comparable with that in HC, but that there is significantly increased production of IL-6 in response to LPS, and particularly increased production of IL-12 following stimulation with LPS and INFγ. We then went on to demonstrate that monocytes of ICOS-deficient patients have significantly increased expression of ICOSL, suggesting that enhanced production of IL-12 might in part result from the failure to downregulate ICOSL. The importance of downregulation of ICOSL by interaction with ICOS as a way of regulating inflammatory responses was previously shown in a murine model. The antigen presenting cells (APCs) of ICOS-transgenic mice (high constitutive expression of ICOS on T cells) showed significant downregulation of ICOSL and consequent inability to generate appropriate cytokine responses [31]. The opposite seems to be true in the case of ICOS-deficient patients, where sustained expression ICOSL expression is associated with prolonged activation of APCs and enhanced production of inflammatory cytokines.

The excessive IL-12 production we have seen in our patients seems to be clinically relevant since this was associated with skewing of helper T cells towards a predominantly Th1 phenotype. A recent study in CVID patients showed that skewed Th1 phenotype of follicular helper cells was associated with immune dysregulation and inflammatory complications [32]. We used findings from our study to select a targeted therapy for one of the patients (P16) with several inflammatory complications (granuloma role of IL-12). Unfortunately, in this case, selective blockade of IL-12 with ustekinumab did not produce the desired outcome. There are number of possible reasons for this, including wrong dose or regimen.

We have also shown reduced CTLA4 surface expression on the effector CD4^+^ T cells and Tregs. Reduced expression of this inhibitory receptor due to heterozygous germline mutations in *CTLA4* is known to cause severe immune dysregulation [33, 34]. It is therefore possible that in ICOS deficiency, in addition to impaired IL-10 production, reduced CTLA4 expression is relevant for the pathogenesis of autoimmune and inflammatory complications. Abatacept (CTLA4-Ig) has been used successfully to treat patients with CTLA4 haploinsufficiency [35] and LRBA deficiency [36], and considering our findings, abatacept might also be a therapeutic option for selective patients with ICOS deficiency.

We thus conclude that *ICOS* still accounts for a small but growing group of patients with combined PID. The clinical phenotypes associated with this genetic diagnosis continue to expand. Immune dysregulation resulting in overlapping inflammatory and immunodeficiency complications is typical for many patients. Improved understanding of this process might lead to improved targeted therapies in the future.

## Electronic Supplementary Material

ESM 1 Gating strategy for Th1 and Th2. **a** T cells were gated based on CD3 positivity and low side scatter. **b** CD4+CD8− helper T cells were then examined for CD45RA and CD27 expression. **c** CD45RA− memory T cells were then segregated into four populations based on CD183 and CD196 expression. **d** Th1 cells were defined as CD183+CD196− and Th2 cells were defined as CD183−CD196− (PNG 1038 kb)

High Resolution (TIFF 9154 kb)

ESM 2 Pedigrees. Patients P16, P17, and P18 from the same ethnic background (Pakistani) but not from the same kindred all carry novel homozygous deletion c.323_332del (p.F108TfsX11). P19 and P20 individual Sanger sequencing results for each patients shown. P21–22 family pedigree and representative Sanger sequencing results. Dots in the individual pedigree represent a carrier status (PNG 1354 kb)

High Resolution (TIFF 9154 kb)

ESM 3 Prediction of pathogenic effects of selected ICOS variants. **a** The consequence of pathogenic missense mutations (p.F119S and p.V151L) conformational change and significant impairment of arbitrary mutation downstream of this position considering multiple sequence alignment, structural features, and solvent accessibility predicted with SNAP2 (a trained classifier based on a machine learning device called “neural network” which distinguishes between effect and neutral variants/non-synonymous SNPs by taking a variety of sequence and variant features into account cross-validation sustained two-state accuracy of 82%, PMID 26110438). **b** Conformational changes in the proteins induced by the novel missense amino acid substitution reported in the ICOS protein (p.F119S and p.V151L) predicted by meta-disorder (MD), protein-protein interaction sites (PPSites), identifying and protein-DNA binding sites (DISIS & SomeNA to be released shortly), and PROFsec regarding secondary structure elements and solvent accessibility using evolutionary information from multiple sequence alignments and a multi-level system (PMID 8066087, PMID 20081223) (PNG 2661 kb)

High Resolution (TIFF 9154 kb)

ESM 4 ICOS expression P16. PBMC was isolated from healthy control (HC) and patient and stimulated at 10^5^ cells/well with PHA or immobilized anti-CD3 (aCD3) as indicated for 3 days at 37 °C, 5% C0_2_. Cell were recovered, washed, and incubated with anti-CD3-PercP, CD4-APC and anti ICOS/HLA-DR-pe. **a** and **b** expression of ICOS/HLA-DR on CD3^+^ CD4^+^ T cells was assessed using a FACSCalibur flow cytometer (BD). The bar charts represent the average of 2 separate experiments. **c** Histogram showing ICOS expression: filled (dark gray peak) with solid lines–isotype staining control; filled (light gray peak) with dotted lines–isotype staining patient; un-filled with solid lines–ICOS staining control; un-filled with dotted lines–ICOS staining patient (PNG 497 kb)

High Resolution (TIFF 9154 kb)

ESM 5 Delayed reaction to antibiotics and lymphocyte transformation assay. **a** Shows cutaneous lesions which had developed after patient took 3 days of doxycycline. Similar reactions were noted with other antibiotics including amoxicillin and ciprofloxacin. **b** PBMC from HC and patient (P16) were cultured sterile conditions; cells were cultured in 96-well plates in the presence of PHA (Sigma, 5–10 μg/ml) or Doxycycline hydrochloride (Doxy), Ciprofloxacin hydrochloride (Cipro), and clarithromycin (Clarithro) (Sigma) and were cultured for 4 days (for PHA) and 7 days (for the antibiotics) with ^3^H-thymidine (0.037 MBq/well) added for the final 16 h of culture. At the end of the culture time, cells were harvested using a cell harvester (Skatron, Norway) and thymidine incorporation assessed following inclusion with 5 ml/well of Optiphase Hisafe 3 scintillant (Perkin Elmer) using a *B* counter (Wallac 1409 DSA liquid scintillation counter). Results are expressed as CPM following a 1 min measurement. The bar charts represent the average of 2 separate experiments (PNG 2985 kb)

High Resolution (TIFF 9154 kb)

ESM 6 (DOCX 12 kb)

ESM 7 (DOCX 24 kb)

ESM 8 (DOCX 76 kb)

## Abbreviations

AAAAI: American Academy of Allergy, Asthma and Immunology
Ab: Antibody
ACMG: American College of Medical Genetics and Genomics
CVID: Common variable immunodeficiency
CTLA4: Cytotoxic T lymphocyte–associated protein 4
ESID: European Society for Immunodeficiencies
HC: Healthy controls
IBD: Inflammatory bowel disease
ICOS: Inducible T cell co-stimulator
ICOSL: ICOS ligand
IFNγ: Interferon gamma
Ig: Immunoglobulin
IL: Interleukin
IUIS: International Union of Immunological Societies
LPS: Lipopolysaccharide
PBMC: Peripheral blood mononuclear cell
PHA: Phytohemagglutinin
Th: Helper T cells
TNF: Tumor necrosis factor
TLR: Toll-like receptor
WES: Whole exome sequencing
