# Supplementary figures and images for: Expanding Clinical Phenotype and Novel Insights into the Pathogenesis of ICOS Deficiency
Source: J Clin Immunol. 2019 Dec 20;40(2):277–88. doi: 10.1007/s10875-019-00735-z (PMC7082411; doi:10.1007/s10875-019-00735-z)

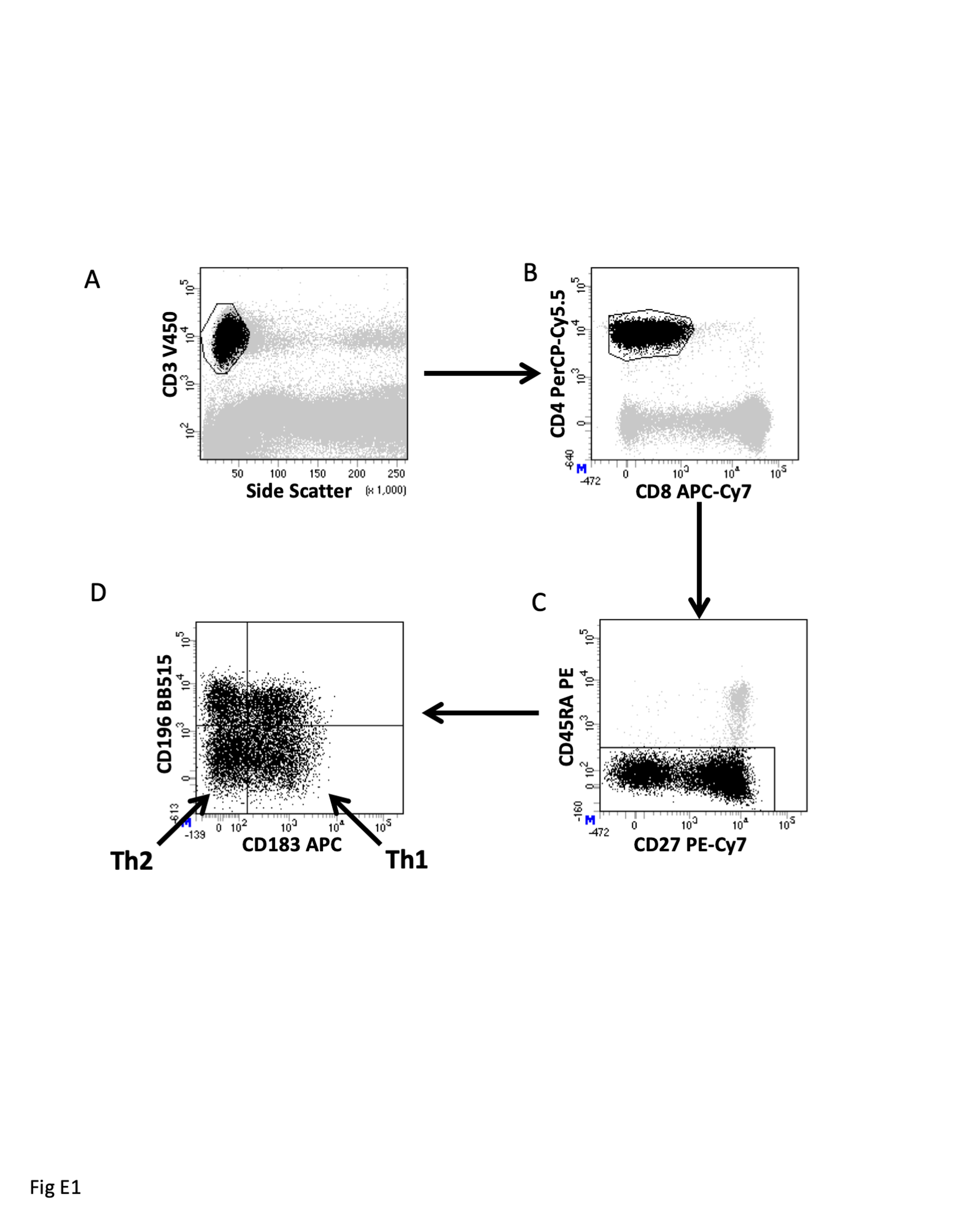

Supplement: Supplementary file 1 — Gating strategy for Th1 and Th2. a T cells were gated based on CD3 positivity and low side scatter. b CD4+CD8− helper T cells were then examined for CD45RA and CD27 expression. c CD45RA− memory T cells were then segregated into four populations based on CD183 and CD196 expression. d Th1 cells were defined as CD183+CD196− and Th2 cells were defined as CD183−CD196− (PNG 1038 kb) [file 10875_2019_735_Fig6_ESM.png]

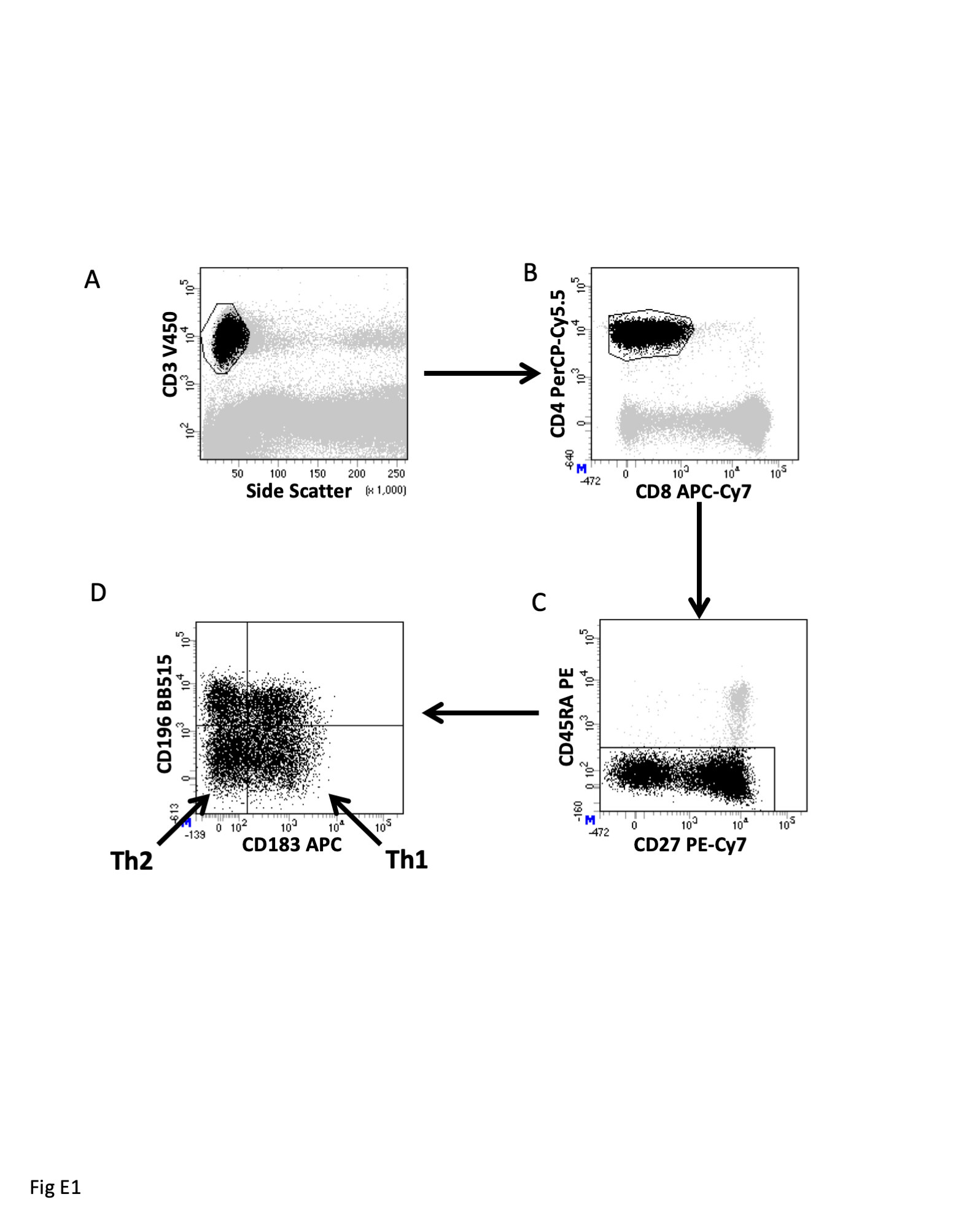

Supplement: Supplementary file 2 — High Resolution (TIFF 9154 kb) [file 10875_2019_735_MOESM1_ESM.tiff]

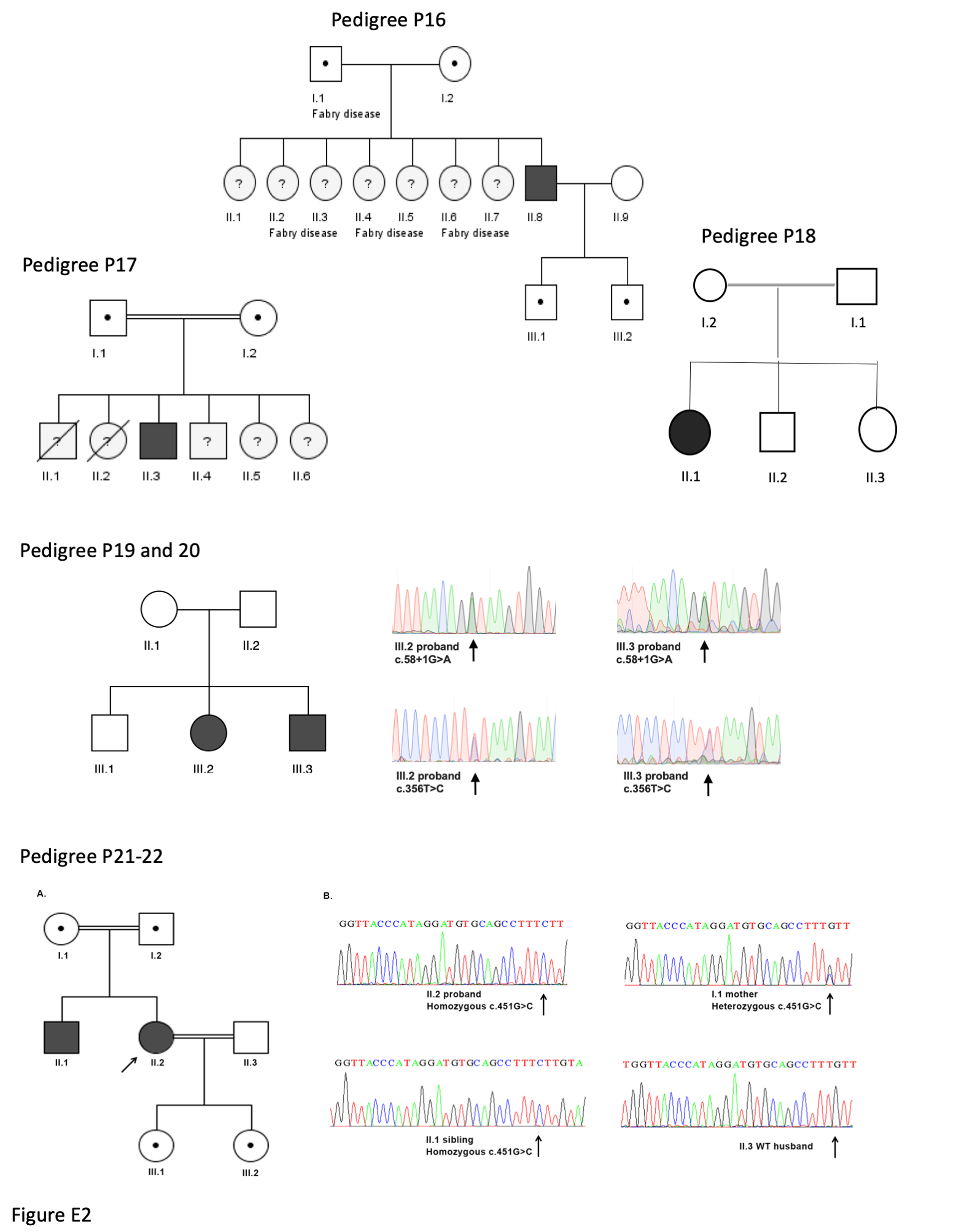

Supplement: Supplementary file 3 — Pedigrees. Patients P16, P17, and P18 from the same ethnic background (Pakistani) but not from the same kindred all carry novel homozygous deletion c.323_332del (p.F108TfsX11). P19 and P20 individual Sanger sequencing results for each patients shown. P21–22 family pedigree and representative Sanger sequencing results. Dots in the individual pedigree represent a carrier status (PNG 1354 kb) [file 10875_2019_735_Fig7_ESM.png]

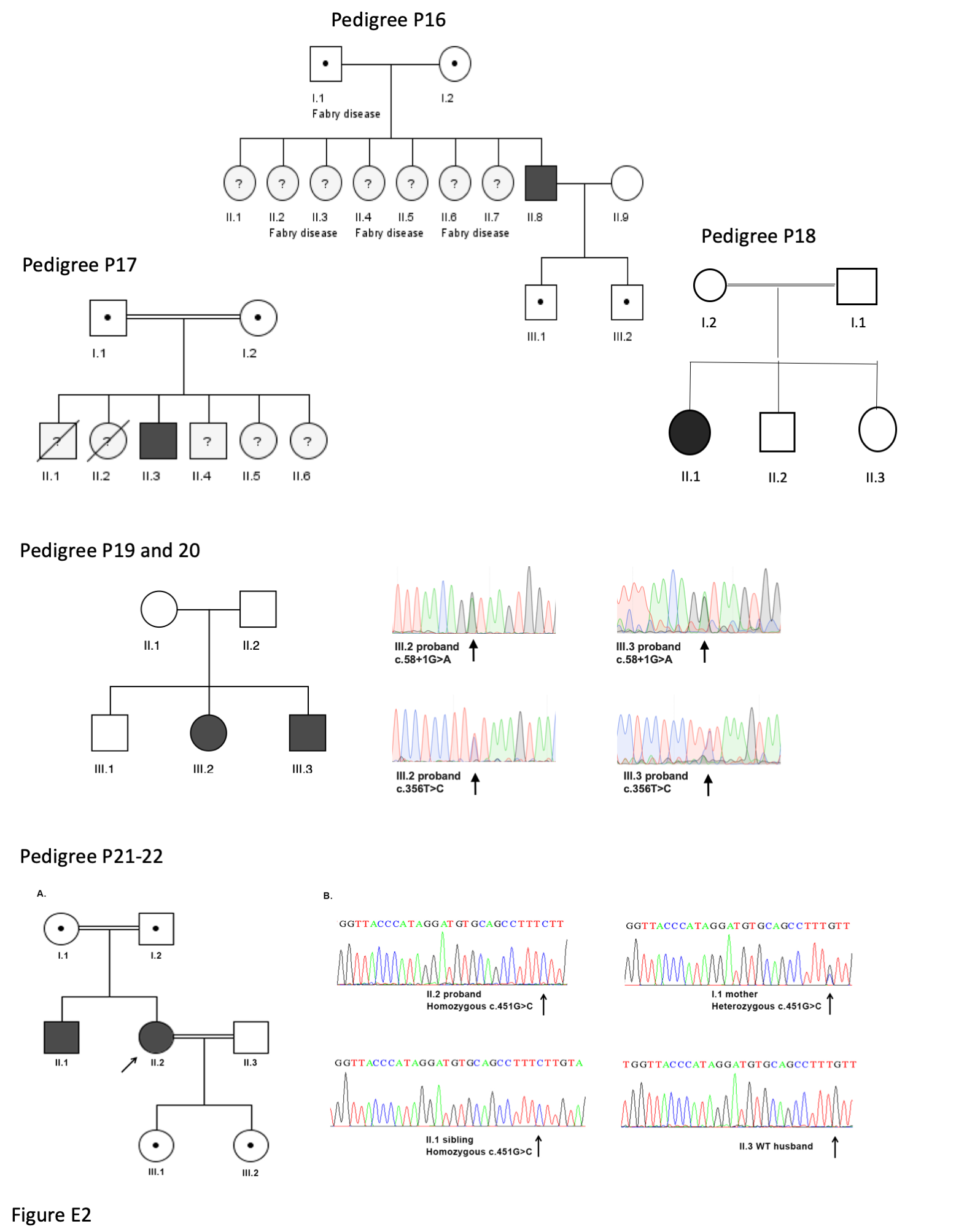

Supplement: Supplementary file 4 — High Resolution (TIFF 9154 kb) [file 10875_2019_735_MOESM2_ESM.tiff]

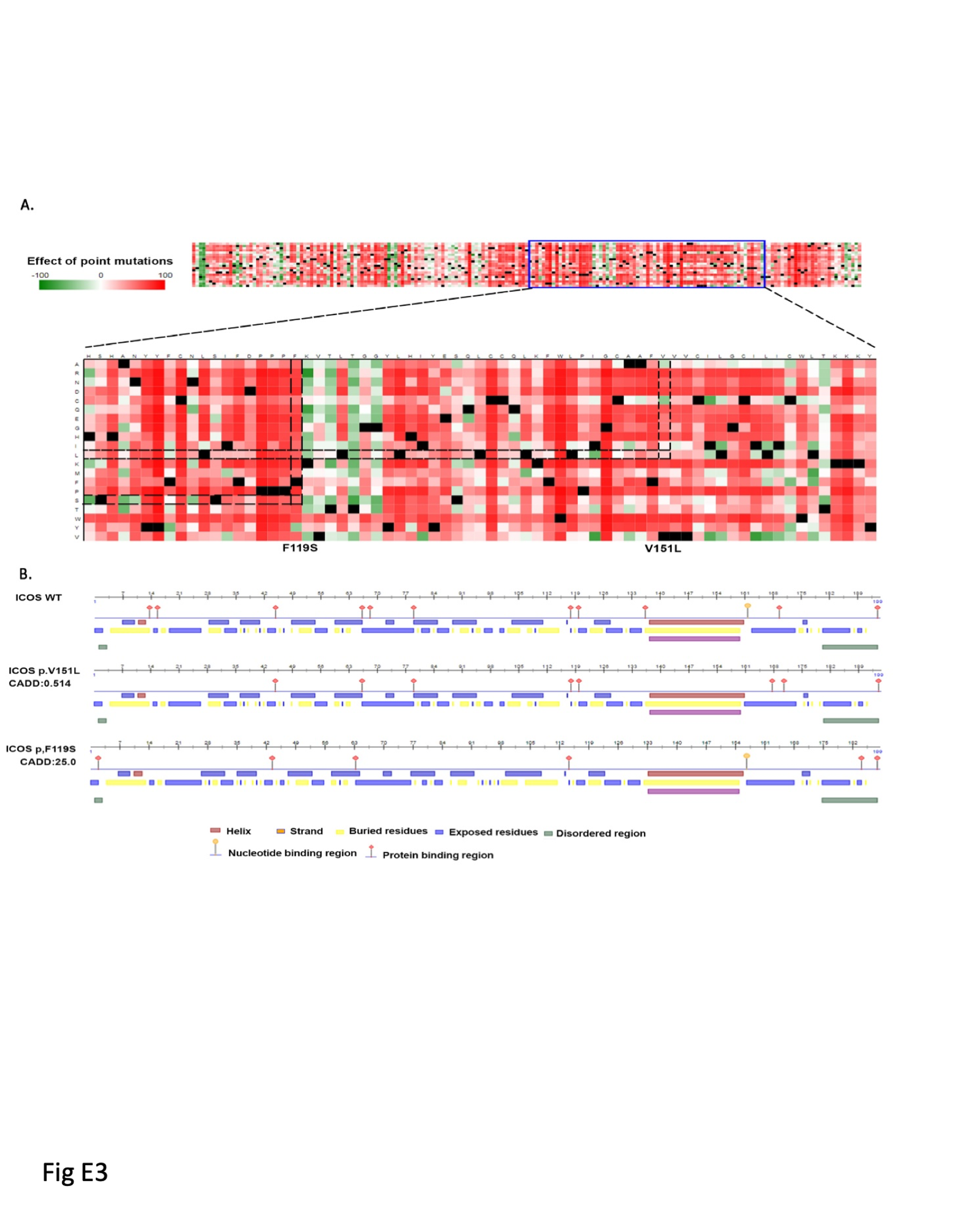

Supplement: Supplementary file 5 — Prediction of pathogenic effects of selected ICOS variants. a The consequence of pathogenic missense mutations (p.F119S and p.V151L) conformational change and significant impairment of arbitrary mutation downstream of this position considering multiple sequence alignment, structural features, and solvent accessibility predicted with SNAP2 (a trained classifier based on a machine learning device called “neural network” which distinguishes between effect and neutral variants/non-synonymous SNPs by taking a variety of sequence and variant features into account cross-validation sustained two-state accuracy of 82%, PMID 26110438). b Conformational changes in the proteins induced by the novel missense amino acid substitution reported in the ICOS protein (p.F119S and p.V151L) predicted by meta-disorder (MD), protein-protein interaction sites (PPSites), identifying and protein-DNA binding sites (DISIS & SomeNA to be released shortly), and PROFsec regarding secondary structure elements and solvent accessibility using evolutionary information from multiple sequence alignments and a multi-level system (PMID 8066087, PMID 20081223) (PNG 2661 kb) [file 10875_2019_735_Fig8_ESM.png]

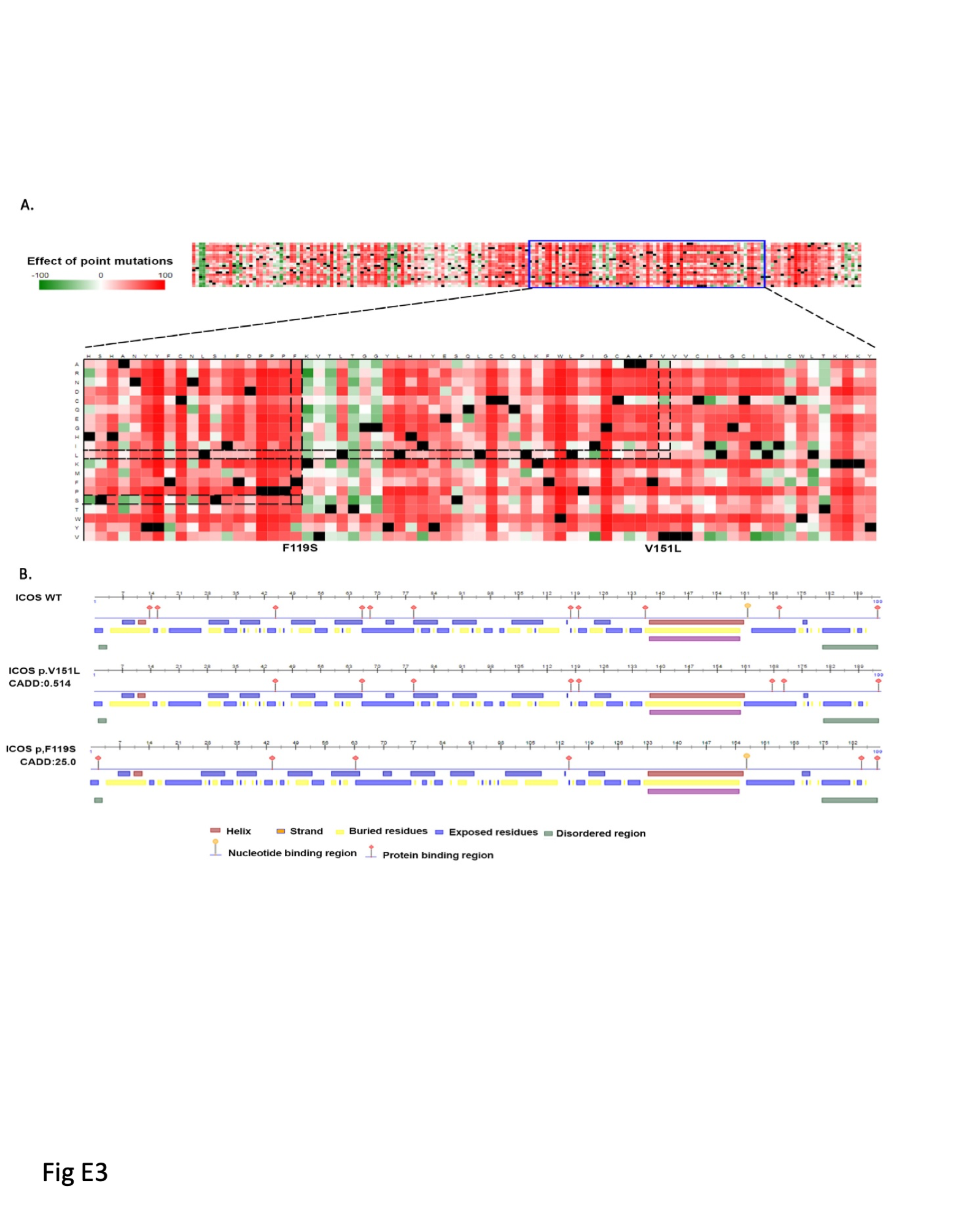

Supplement: Supplementary file 6 — High Resolution (TIFF 9154 kb) [file 10875_2019_735_MOESM3_ESM.tiff]

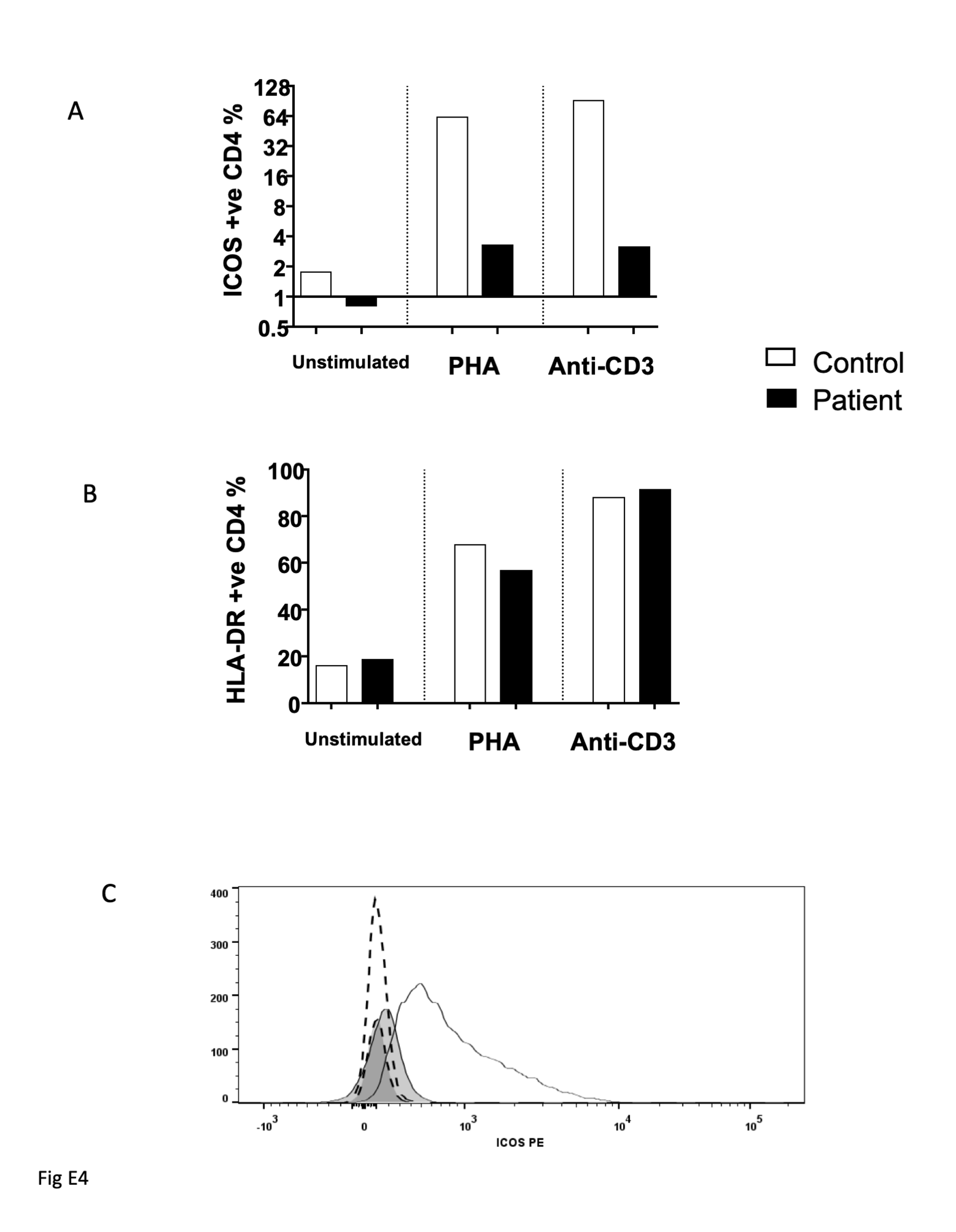

Supplement: Supplementary file 7 — ICOS expression P16. PBMC was isolated from healthy control (HC) and patient and stimulated at 105 cells/well with PHA or immobilized anti-CD3 (aCD3) as indicated for 3 days at 37 °C, 5% C02. Cell were recovered, washed, and incubated with anti-CD3-PercP, CD4-APC and anti ICOS/HLA-DR-pe. a and b expression of ICOS/HLA-DR on CD3+ CD4+ T cells was assessed using a FACSCalibur flow cytometer (BD). The bar charts represent the average of 2 separate experiments. c Histogram showing ICOS expression: filled (dark gray peak) with solid lines–isotype staining control; filled (light gray peak) with dotted lines–isotype staining patient; un-filled with solid lines–ICOS staining control; un-filled with dotted lines–ICOS staining patient (PNG 497 kb) [file 10875_2019_735_Fig9_ESM.png]

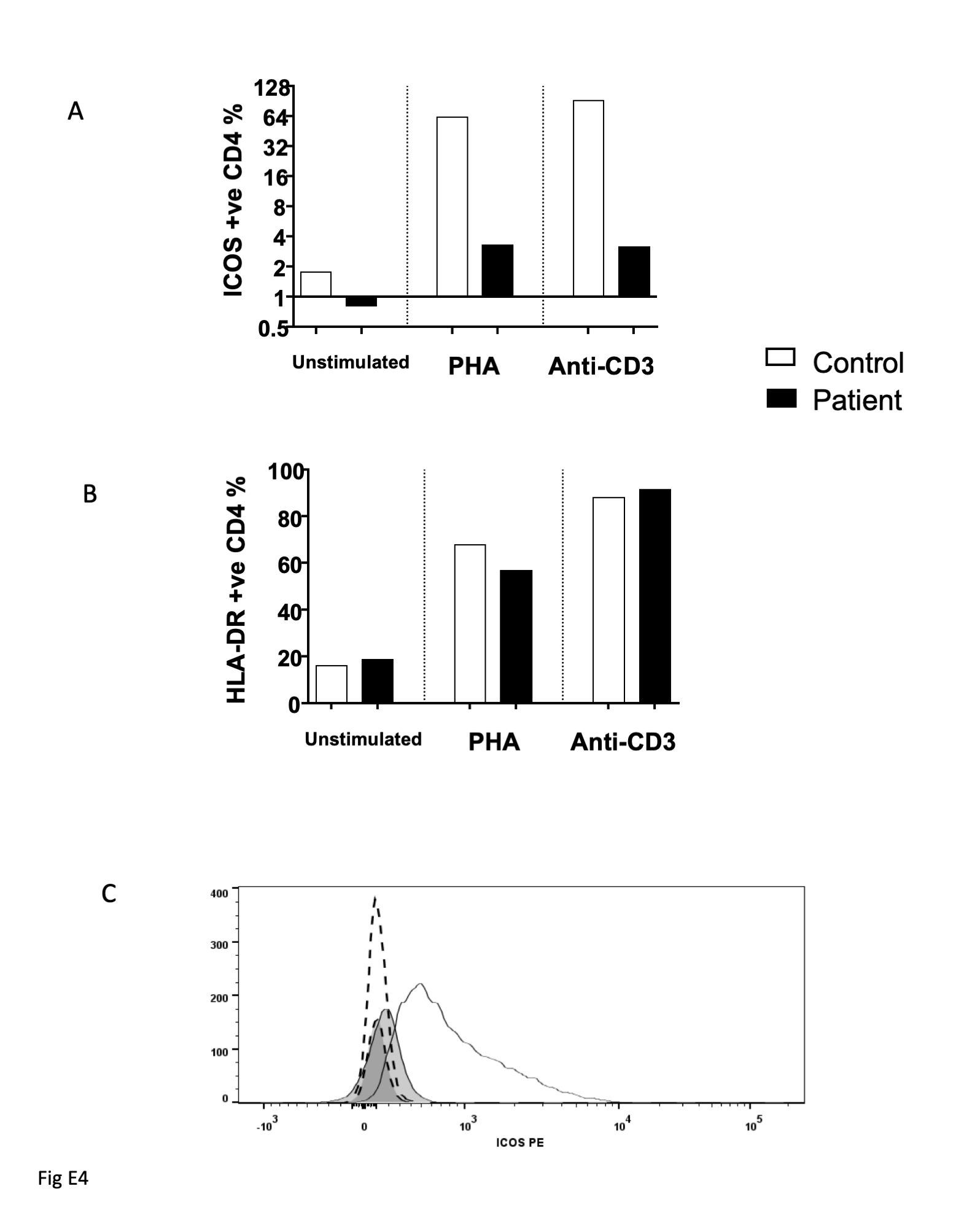

Supplement: Supplementary file 8 — High Resolution (TIFF 9154 kb) [file 10875_2019_735_MOESM4_ESM.tiff]

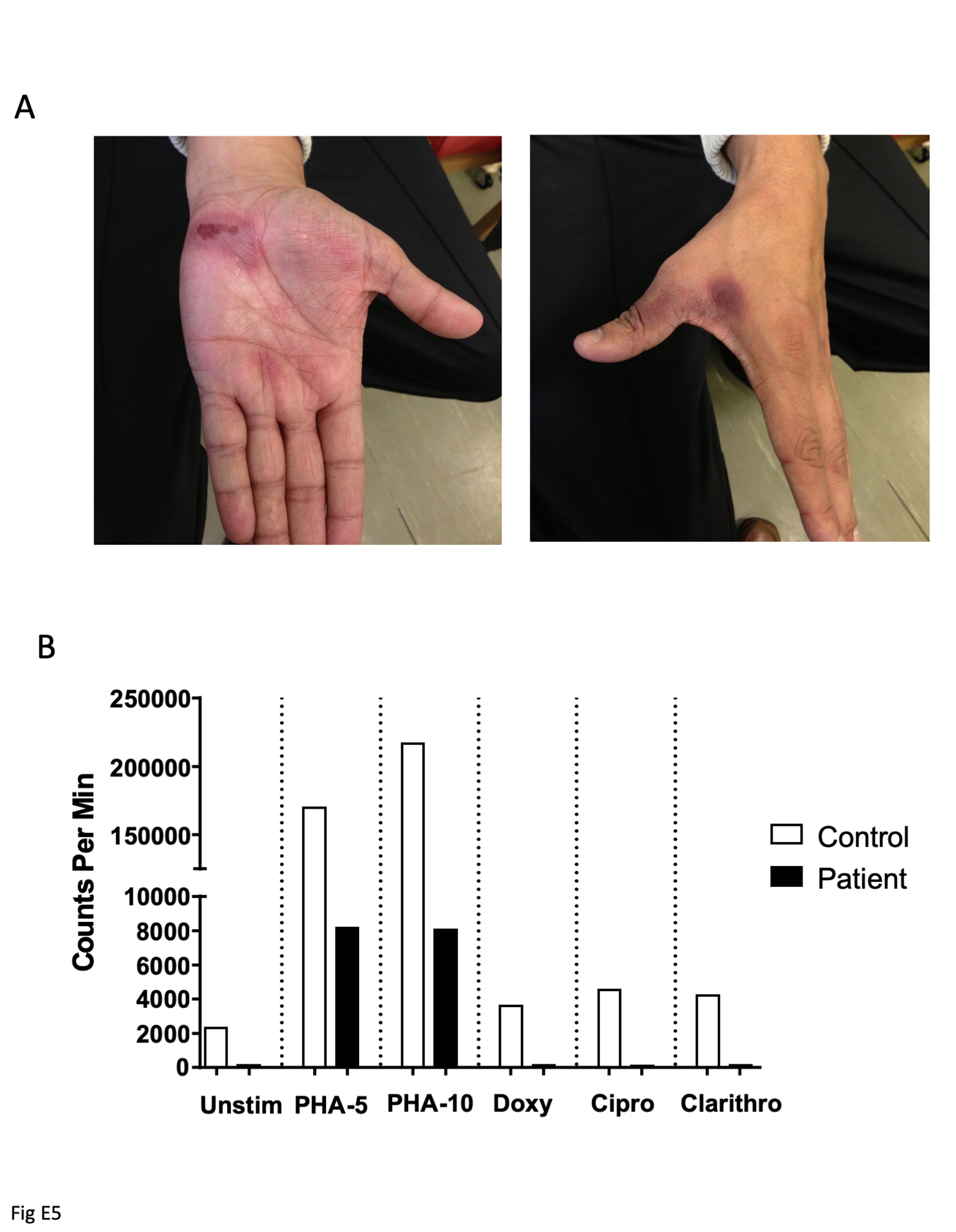

Supplement: Supplementary file 9 — Delayed reaction to antibiotics and lymphocyte transformation assay. a Shows cutaneous lesions which had developed after patient took 3 days of doxycycline. Similar reactions were noted with other antibiotics including amoxicillin and ciprofloxacin. b PBMC from HC and patient (P16) were cultured sterile conditions; cells were cultured in 96-well plates in the presence of PHA (Sigma, 5–10 μg/ml) or Doxycycline hydrochloride (Doxy), Ciprofloxacin hydrochloride (Cipro), and clarithromycin (Clarithro) (Sigma) and were cultured for 4 days (for PHA) and 7 days (for the antibiotics) with 3H-thymidine (0.037 MBq/well) added for the final 16 h of culture. At the end of the culture time, cells were harvested using a cell harvester (Skatron, Norway) and thymidine incorporation assessed following inclusion with 5 ml/well of Optiphase Hisafe 3 scintillant (Perkin Elmer) using a B counter (Wallac 1409 DSA liquid scintillation counter). Results are expressed as CPM following a 1 min measurement. The bar charts represent the average of 2 separate experiments (PNG 2985 kb) [file 10875_2019_735_Fig10_ESM.png]

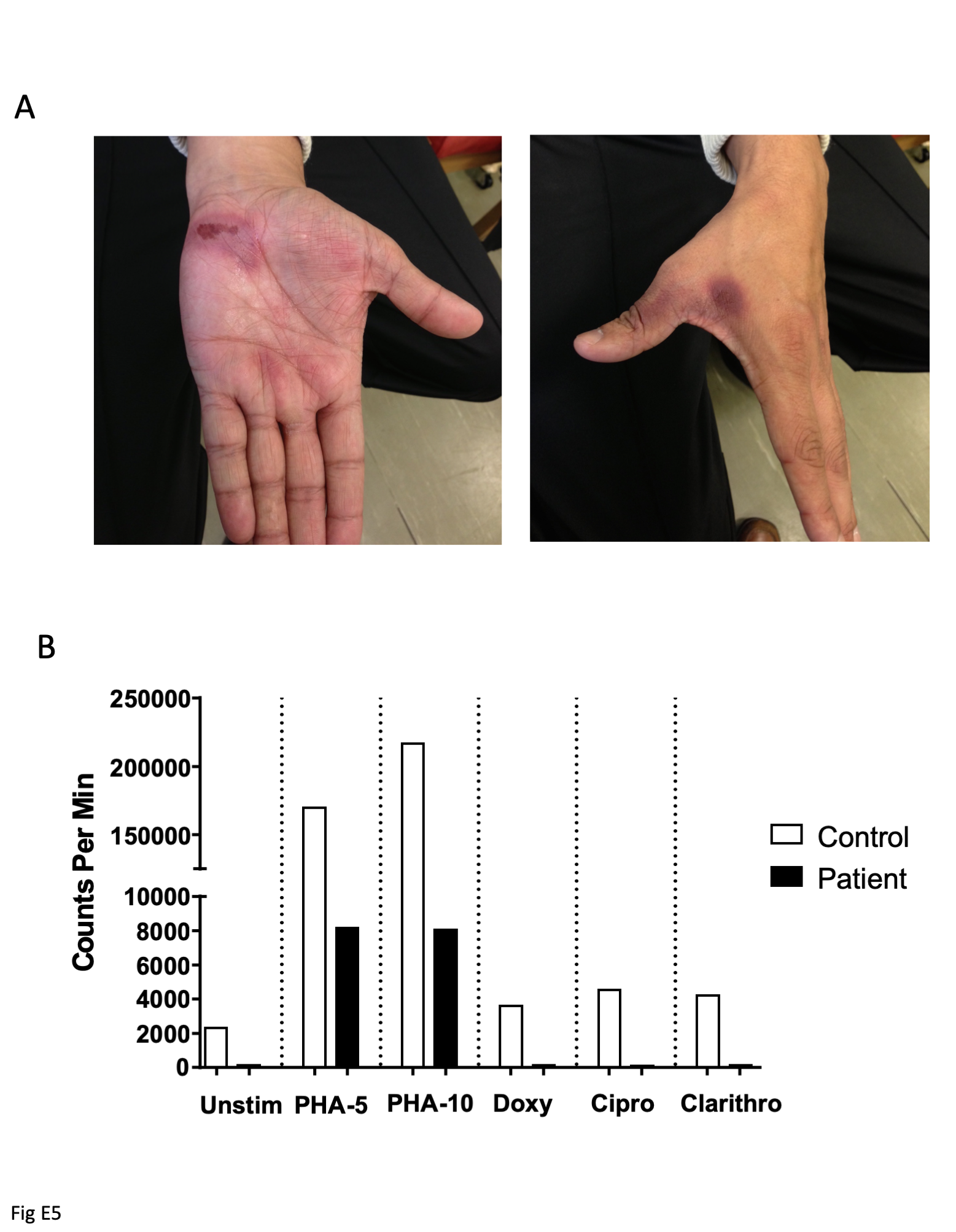

Supplement: Supplementary file 10 — High Resolution (TIFF 9154 kb) [file 10875_2019_735_MOESM5_ESM.tiff]
