# Supplementary material for: Expanding Clinical Phenotype and Novel Insights into the Pathogenesis of ICOS Deficiency
Source: J Clin Immunol. 2019 Dec 20;40(2):277–88. doi: 10.1007/s10875-019-00735-z (PMC7082411; doi:10.1007/s10875-019-00735-z)
Supplement: Supplementary file 11 — (DOCX 12 kb) [file 10875_2019_735_MOESM6_ESM.docx]

**Supplementary Methods**

Antibodies used: The following antibody clones were used in this study. CD303 (AC144; Miltenyi Biotec), CD123 (AC145; Miltenyi Biotec), HLA-DR (AC122; Miltenyi Biotec), CD141 (AD5-14H12; Miltenyi Biotec), CD64 (10.1.1; Miltenyi Biotec), CD3 (BW264/56; Miltenyi Biotec), CD19 (LT19; Miltenyi Biotec), CD4 (SK3; Miltenyi Biotec) CD11c (B-ly6; BD Biosciences), CD14 (TÜK4 and MφP9; BD Biosciences); CD16 (3G8; BD Biosciences), CD56 (B159; BD Biosciences), ICOS (xxx; BD Biosciences), ICOSL (xxx ; BD Biosciences); CD38 (DX9; Biolegend) and CD1c (L161; Biolegend), CD3 V450 (UCHT1; BD Horizon), CD196 BB515 (11A9; BD Horizon), CD45RA PE (ALB11; IOTest), CD4 PerCP-Cy5.5 (SK3; BD Biosciences), CD27 PE-Cy7 (M-T271; BD Horizon), CD183 APC (1C6; BD Pharmingen), CD185 APC-R700 (RF8B2; BD Pharmingen), CD8 APC-Cy7 (SK1; BD Biosciences).

CTLA-4 expression experiments:

CD3-V500, clone UCHT1, CD4-BV421, clone RPA T4, CD25 PeCy7, clone 2A3, CD127 PERPCP-cy5.5, CTLA-4 (CD152, antibody clone BNI3) all from (Becton Dickinson).
