# Supplementary material for: Expanding Clinical Phenotype and Novel Insights into the Pathogenesis of ICOS Deficiency
Source: J Clin Immunol. 2019 Dec 20;40(2):277–88. doi: 10.1007/s10875-019-00735-z (PMC7082411; doi:10.1007/s10875-019-00735-z)
Supplement: Supplementary file 12 — (DOCX 24 kb) [file 10875_2019_735_MOESM7_ESM.docx]

**Supplementary Results**

**Clinical vignettes**

**P16**

35 year old male of Pakistani origin presented with an 8 year history of recurrent sinus infections, panhypogammaglobulinemia and abnormal liver function test (LFTs) following a recent episode of pneumonia. Further evaluation revealed absent vaccine response, reduced proliferation response to PHA and mild to moderate lymphopenia affecting all subsets except CD4 T-cells. His 4 sisters, parents and two sons do not report any symptoms suggestive of immunodeficiency. However, father and 3 sisters have a genetic diagnosis of Fabry’s disease. Ig replacement therapy was intravenously started for a presumed diagnosis of CVID. There were incidence of unusual infections including persistent *Molluscum contagiosum*, resistant warts requiring inta-lesional bleomycin three years after diagnosis and dermatophytosis requiring prolonged topical antifungals eight years after diagnosis.

He also reported a 12-month history of reacting to various oral antibiotics including amoxicillin, erythromycin, septrin and augmentin. Patch testing was negative to all these above antibiotics. Oral provocation testing with moxicillin, doxycyclineand ciprofloxacin was tolerated without any immediate reactions but consistently induced desquamation of the hands and feet 48 – 72 hours later. In vitro drug proliferation assays did not reveal any significant proliferation in response to the antibiotics.

The patient experienced intermittent diarrheal episodes whilst on immunoglobulin replacement and was investigated twice. Initial investigations a year after starting treatment did not reveal any significant abnormalities on endoscopies but subsequent investigations 7 years later showed inflammatory cell infiltrate of the lamina propria with focal cryptitis and crypt abscess formation of the colon. Faecal elastase was normal and negative SeHCAT scan excluded bile salt malabsorption.

Screening for autoimmune and metabolic liver disorders as well as CT imaging of the liver and biliary tract did not reveal any cause for the persistently elevated alanine aminotransferase (ALT) and alkaline phosphatase. Liver biopsy revealed early sclerosing cholangitis with portal tract inflammation and ductopenia but no evidence of fibrosis. Magnetic resonance cholangiopancreatography (MRCP) was unremarkable. The elevated liver enzymes responded to oral ursodeoxycholic acid (UDCA).

Review of the haematological system revealed persistent leucopenia and thrombocytopenia, splenomegaly and low grade retroperitoneal and pelvic lymphadenopathy. Bone marrow showed increased infiltration with cytotoxic T-cells, hyperplastic haemopoiesis and large lymphoid aggregates with small granulomata. Due to worsening cytopenias immunosuppression was attempted with oral prednisolone without any significant response. In vitro assays showed increased IL12 production and therefore Ustekinumab therapy to reverse the inflammatory cytokine milieu was attempted without much success. Currently the patient is being assessed for the possibility of HSCT.

**P17**

39 year old male of Pakistani origin presented with life long history of recurrent chest, ear and throat infections. There was history of consanguinity with parents being first cousins. One of his sisters died during infancy and a brother with history of recurrent infections died with liver disease as a 17 year old. He also has three other healthy siblings – 1 brother and 2 sisters. Investigations revealed panhypogammaglobulinemia, B-cell lymphopenia and absent vaccine response to polysaccharide and protein antigens. On presentation, there was no history of atypical infections, cytopenia, gut or liver disease. High resolution computed tomography of the chest showed non-specific bronchial wall thickening but no evidence of bronchiectasis. The patient kept refusing immunoglobulin replacement therapy for three-and-a-half years after diagnosis.

A year after diagnosis, he developed cold abscess of the right arm with regional lymphadenopathy requiring drainage and six months of anti-tuberculous therapy. The patient developed impaired LFTs around this time which progressed over the next couple of years. Further investigations showed predominant cholestatic picture and biopsy showed chronic hepatitis with portal and sinusoidal lymphocytic infiltrates. There was no evidence of biliary disease on histology or imaging. Apart from mild splenomegaly and low grade retroperitoneal lymphadenopathy, imaging of the liver was unremarkable without portal hypertension. The patient agreed to start immunoglobulin replacement few months later due to on-going recurrent chest and sinus infections. However, he would miss two to three months of treatment every year due to prolonged trips to his native country. A diagnosis of ICOS deficiency was made following sequencing 13 years after initial presentation. Liver biopsy was repeated due to worsening LFTs, which showed moderately severe lobular hepatitis, some loss of bile ducts, mild, increase in portal fibrosis but no bridging fibrosis. Ursodeoxycholic acid (UDCA) therapy was initiated. Gastroscopy was done to exclude varices, which showed candidiasis. A year later there was further worsening of LFTs which led to repeat biopsy that showed similar degree and pattern of inflammation but worsening ductopenia and new cholestasis. Imaging of the liver showed coarse parenchyma and nodular capsule suggestive of cirrhosis. The patient was treated with oral prednisolone.

The patient travelled to Pakistan whilst on Prednisolone and was admitted on return with diarrhoea and Salmonella sepsis complicated by disseminated intravascular coagulation. The infection initially responded to antibiotics but developed worsening of the liver function and also acquired pneumonia. He unfortunately did not survive this episode, which was a combination of decompensated liver disease, hospital acquired pneumonia and pulmonary edema.

**P18**

Presented aged 3 with a lobar pneumonia requiring intravenous antibiotic therapy in hospital.  Her previous history was notable for neonatal (10 days) RSV bronchiolitis with a lobar pneumonia and frequent upper respiratory tract infections thereafter, but growth and development were normal.  She was found to have undetectably low IgG and low but not absent IgM and IgA; total B cell number was normal, but with an overwhelmingly naïve B cell phenotype.  She responded very well to immunoglobulin therapy and cotrimoxazole prophylaxis but did require hospitalisation for intercurrent viral infections including parechovirus gastroenteritis.  Owing to adverse outcome in an affected adult relative, the family elected for hematopoietic stem cell transplantation from a haploidentical parent when the patient was 5 years of age.  This was uneventful and led to 100% donor chimerism and good immune reconstitution to date (8 month follow up; off immunoglobulin, pending revaccination).

**P19**

is a 34-year-old male who presented at six years of age with pneumonia requiring intensive care. He did not suffer from recurrent infections but developed with weight loss and diarrhoea around 10 years of age. Coeliac disease was diagnosed on small bowel biopsy and his symptoms are controlled on a gluten-free diet. Low IgG was initially identified as an incidental finding around the age of 20 years; IgA and IgM levels were both subsequently reduced. He was subsequently diagnosed with common variable immunodeficiency and commenced immunoglobulin replacement therapy. He had low switch memory B cells, low total B cell numbers with normal T cell numbers and T cell proliferation assays. His family history was notable for an older sister with severe juvenile idiopathic arthritis and hypogammaglobulinaemia. His parents are alive, his mother has type II diabetes and pancreatic sufficiency. He has a younger brother who is well. He was screened by whole genome sequencing and compound heterozygous mutation in ICOS identified. He has been well in adult hood with approximately one respiratory tract infection per year requiring antibiotics and one episode of giardia gastrointestinal infection that responded to metronidazole. A CT chest at the age of 32 identified early bronchiectasis in two lobes, with normal lung function. He has Gilbert’s syndrome.

**P20**

is the 36-year-old sister of P19. She presented with juvenile arthritis the age of 18 months, developed inflammatory bowel disease at the age of 12 and developed psoriasis in childhood. She required multiple immunosuppressive agents including methotrexate and gold and is maintained on long-term low-dose oral prednisolone (5mg daily) and mesalazine. Despite treatment she developed severe joint deformities in all limbs and growth retardation and required hip replacement page 26 years. Hypogammaglobulinaemia was subsequently detected incidentally aged 22 without a history of infections. She had normal IgA and IgM; vaccine responses were low at baseline to tetanus, Haemophilus influenza and pneumococcus but vaccination challenge was not performed due to a very low IgG level (<3g/L). She had mild lymphocytosis with low percentage of total B cells and switched memory B cells. Introduction of immunoglobulin treatment resulted in a severe reaction to two different products, with a florid extensive rash that required oral prednisolone treatment and resulted in termination of immunoglobulin replacement. Allergy testing with intradermal immunoglobulin products confirmed delayed hypersensitivity reaction to all ten products tested. She remains off immunoglobulin treatment without significant infections but bronchiectasis affecting single lobe on a recent CT scan. She has developed genital warts. She was confirmed to have the same compound heterozygous mutation in ICOS as her brother.

**P21-22**

The P21 is an Iranian female born to consanguineous parent and completed her scheduled vaccinations without complication without family history of primary immunodeficiency. She presented at three years of age with recurrent sinusitis. At age 6 she developed otitis media and at age 15 pneumonia. Currently, at age 38 she suffers from chronic enteritis. Immunologic assessment at age 33 revealed low serum immunoglobulins, normal lymphocyte subsets and poor response to tetanus and diphtheria booster vaccinations (**Table E7**). Detailed immunologic profiling of the patient was compatible with Euroclass smB^-^Tr^hi^21^lo^ and pattern 4 B-cell development defects (germinal center defect) based on the standard CVID classifications [^35^](#_ENREF_35)^,^ [^36^](#_ENREF_36). Thus, CVID was diagnosed. Whole exome sequencing (WES) was performed and, after exclusion of all genes associated with primary immunodeficiency [^28^](#_ENREF_28), revealed a novel homozygous missense mutation c.451G>C (allele frequency in 60,706 unrelated individuals within the Exome Aggregation Consortium is 0.0007) in exon 3 of the *ICOS* gene which would result in p.V151L at a highly evolutionary conserved residue within the transmembrane domain of the protein, which is predicted to be probably damaging based on the mutation significance cutoff analysis by the deconformation of the protein and nucleotide binding sites around helical domain of the protein. Sanger sequencing confirmed the mutation in the proband and subsequently in her sibling with a history of chronic sinusitis. The parents and her children were heterozygous for this mutation (**FigE 1**). Subsequent immunologic investigation of the homozygous sibling (P22) revealed a mild pan-hypogammaglobulinemia but normal lymphocyte subsets. Production of specific antibodies after immunization with polypeptides and polysaccharides antigens was also normal (**Table E7)**
