# Supplementary material for: Expanding Clinical Phenotype and Novel Insights into the Pathogenesis of ICOS Deficiency
Source: J Clin Immunol. 2019 Dec 20;40(2):277–88. doi: 10.1007/s10875-019-00735-z (PMC7082411; doi:10.1007/s10875-019-00735-z)
Supplement: Supplementary file 13 — (DOCX 76 kb) [file 10875_2019_735_MOESM8_ESM.docx]

***Table E1*.** Primers used for confirmatory sequencing

| **Patients** | **Exon** | **Tm** | **Forward** | **Reverse** |
| --- | --- | --- | --- | --- |
| P16-18 | EX2 | 60C | TGGAGGGAGAATGTGAAGCTG | GCATCTAAGTGAACTCCAACACA |
| P19-20 | EX1 | 60C | ACTCCCAGAAAACCCACTTCC | CCAGTCCAAATGCCAGAGCT |
| P19-20 | EX2 | 60C | TGGAGGGAGAATGTGAAGCTG | GCATCTAAGTGAACTCCAACACA |
| P21-22 | EX3 | 60C | TGTGGTTCAGCAGAATTTTTCA | GCCATGTGTACTTTTGATGAGG |

| ***Table E2.*** The 268 genes excluded in studied patients using next generation sequencing | | | | | |
| --- | --- | --- | --- | --- | --- |
| **Genes symbols** | | | | | |
| *ACP5* | *CD81* | *GFI1* | *MASP1* | *RNF168* | *TREX1* |
| *ACTB* | *CD8A* | *GUCY2C* | *MASP2* | *RORC* | *TRNT1* |
| *ADA* | *CEBPE* | *HAX1* | *MCM4* | *RNF31* | *TPP1* |
| *ADAM17* | *CFB* | *HPS1* | *MEFV* | *RPSA* | *TPP2* |
| *ADAR* | *CFD* | *HPS4* | *MRE11A* | *RTEL1* | *TTC37* |
| *AICDA* | *CFH* | *HPS6* | *MSH6* | *SAMHD1* | *TTC7A* |
| *AIRE* | *CFHR1* | *IFIH1* | *MS4A1* | *SBDS* | *TYK2* |
| *AK2* | *CFHR2* | *IFNG* | *MTHFD1* | *SEMA3E* | *UNC119* |
| *AP3B1* | *CFHR3* | *IFNGR1* | *MVK* | *SERPING1* | *UNC13D* |
| *APOL1* | *CFHR4* | *IFNGR2* | *MYD88* | *SH2D1A* | *UNC93B1* |
| *ATM* | *CFHR5* | *IGLL1* | *NBN* | *SH3BP2* | *UNG* |
| *B2M* | *CFI* | *IKBKB* | *NCF1* | *SKIV2L* | *USB1* |
| *BLM* | *CFP* | *IKBKG* | *NCF2* | *SCL29A3* | *VPS13B* |
| *BLNK* | *CHD7* | *IKZF1* | *NCF4* | *SLC35C1* | *VPS45* |
| *BTK* | *CIITA* | *IL10* | *NFAT5* | *SLC37A4* | *WIPF1* |
| *C1R* | *CLPB* | *IL10RA* | *NFKBIA* | *SLC46A1* | *XIAP* |
| *C1QA* | *CLEC7A* | *IL10RB* | *NFKB2* | *SMARCAL1* | *ZAP70* |
| *C1QB* | *COLEC11* | *IL12B* | *NHP2* | *SP110* | *ZBTB24* |
| *C1QC* | *CORO1A* | *IL12RB1* | *NLRC4* | *STAT1* |  |
| *C1S* | *CR2* | *IL17F* | *NLRP3* | *STAT2* |  |
| *C2* | *CSF2RA* | *IL17RA* | *NLRP12* | *STAT3* |  |
| *C3* | *CSF3R* | *IL17RC* | *NOD2* | *STAT5B* |  |
| *C4A* | *COH1* | *IL1RN* | *NOD10* | *STIM1* |  |
| *C4B* | *COPA* | *IL21* | *NRAS* | *STK4* |  |
| *C5* | *CORO1A* | *IL21R* | *ORAI1* | *STX11* |  |
| *C6* | *CTLA4* | *IL2RA* | *PARN* | *SPINK5* |  |
| *C7* | *CTPS1* | *IL2RG* | *PIK3CD* | *STXBP2* |  |
| *C8A* | *CTSC* | *IL36RN* | *PIK3R1* | *TAP1* |  |
| *C8B* | *CXCR4* | *IL6* | *PLCG2* | *TAP2* |  |
| *C8G* | *CYBA* | *IL7R* | *PMS2* | *TAPBP* |  |
| *C9* | *CYBB* | *INO80* | *PNP* | *TAZ* |  |
| *CARD9* | *DCLRE1B* | *IRAK4* | *POLE* | *TBK1* |  |
| *CARD11* | *DCLRE1C* | *IRF7* | *PRF1* | *TBX1* |  |
| *CARD14* | *DKC1* | *IRF8* | *PRKCD* | *TCF3* |  |
| *CASP10* | *DNMT3B* | *ISG15* | *PRKDC* | *TCN2* |  |
| *CASP8* | *DOCK8* | *ITCH* | *PSMB8* | *TERT* |  |
| *CCBE1* | *ELANE* | *ITGB2* | *PSTPIP1* | *THBD* |  |
| *CD17* | *EPG5* | *ITK* | *PTPRC* | *TICAM1* |  |
| *CD19* | *FADD* | *JAGN1* | *RAB27A* | *TINF2* |  |
| *CD27* | *FAS* | *JAK3* | *RAC2* | *TLR3* |  |
| *CD247* | *FASLG* | *KRAS* | *RAG1* | *TMC6* |  |
| *CD3D* | *FCGR3A* | *LAMTOR2* | *RAG2* | *TMC8* |  |
| *CD3E* | *FCN3* | *LCK* | *RBCK1* | *TMEM173* |  |
| *CD3G* | *FERMT1* | *LIG4* | *RFX5* | *TNFRSF13B* |  |
| *CD40* | *FERMT3* | *LPIN2* | *RFXANK* | *TNFRSF13C* |  |
| *CD40LG* | *FOXN1* | *LRBA* | *RFXAP* | *TNFRSF1A* |  |
| *CD46* | *FOXP3* | *LYST* | *RHOH* | *TNFRSF4* |  |
| *CD59* | *FPR1* | *MAGT1* | *RNASEH2A* | *TNFSF12* |  |
| *CD79A* | *G6PC3* | *MALT1* | *RNASEH2B* | *TRAF3* |  |
| *CD79B* | *GATA2* | *MAP3K14* | *RNASEH2C* | *TRAF3IP2* |  |

**Table E3.** Demographic and general data on 22 patients with ICOS deficiency

| **Patient ID** | **Sex** | **Ethnicity** | **Family** | **Zygocity** | **Mutation** | **Protein change** | **Age of onset (year)** | **Age of diagnosis (year)** | **Ref** |
| --- | --- | --- | --- | --- | --- | --- | --- | --- | --- |
| P1 | F | Caucasian  Danube region | F1 | Homozygous | g.18169  1,815 bp deletion | p.T19fsX9 | 27 | 28 | [^12^](#_ENREF_12)^,^ [^14^](#_ENREF_14)^,^ [^33^](#_ENREF_33) |
| P2 | M | Caucasian  Danube region | F1 | Homozygous | g.18169  1,815 bp deletion | p.T19fsX9 | 19 | 21 | [^12^](#_ENREF_12)^,^ [^14^](#_ENREF_14)^,^ [^33^](#_ENREF_33) |
| P3 | M | Caucasian  Danube region | F2 | Homozygous | g.18169  1,815 bp deletion | p.T19fsX9 | 6 | 31 | [^12^](#_ENREF_12)^,^ [^14^](#_ENREF_14)^,^ [^33^](#_ENREF_33) |
| P4 | M | Caucasian  Danube region | F2 | Homozygous | g.18169  1,815 bp deletion | p.T19fsX9 | 12 | 22 | [^12^](#_ENREF_12)^,^ [^14^](#_ENREF_14)^,^ [^33^](#_ENREF_33) |
| P5 | M | Caucasian  Danube region | F3 | Homozygous | g.18169  1,815 bp deletion | p.T19fsX9 | 20 | 38 | [^13^](#_ENREF_13)^,^ [^14^](#_ENREF_14) |
| P6 | M | Caucasian  Danube region | F3 | Homozygous | g.18169  1,815 bp deletion | p.T19fsX9 | 12 | 41 | [^13^](#_ENREF_13)^,^ [^14^](#_ENREF_14) |
| P7 | F | Caucasian  Danube region | F4 | Homozygous | g.18169  1,815 bp deletion | p.T19fsX9 | 8 | 15 | [^13^](#_ENREF_13)^,^ [^14^](#_ENREF_14) |
| P8 | F | Caucasian  Danube region | F4 | Homozygous | g.18169  1,815 bp deletion | p.T19fsX9 | Asymptomatic | 4 | [^13^](#_ENREF_13)^,^ [^14^](#_ENREF_14) |
| P9 | M | Caucasian  Danube region | F4 | Homozygous | g.18169  1,815 bp deletion | p.T19fsX9 | 1.5 | 1.5 | [^13^](#_ENREF_13)^,^ [^14^](#_ENREF_14) |
| P10 | F | Japanese | F5 | Homozygous | c.285 delT | p.F95fsX26 | Infancy | 34 | [^15^](#_ENREF_15) |
| P11 | M | Japanese | F5 | Homozygous | c.285 delT | p.F95fsX26 | 35 | 35 | [^15^](#_ENREF_15) |
| P12 | M | Kuwaiti | F6 | Homozygous | c.90 delG | p.M30IfsX26 | 0.1 | 0.5 | [^17^](#_ENREF_17) |
| P13 | F | Kuwaiti | F6 | Homozygous | c.90 delG | p.M30IfsX26 | 3 | 3 | [^17^](#_ENREF_17) |
| P14 | F | Pakistani | F7 | Homozygous | c.321_330 del | p.F108YfsX10 | 2 | 3.5 | [^16^](#_ENREF_16) |
| P15 | M | Pakistani | F7 | Homozygous | c.321_330 del | p.F108YfsX10 | 2 | 2 | [^16^](#_ENREF_16) |
| P16 | M | Pakistani | F8 | Homozygous | c.323_332del | p.F108YfsX11 | 27 | 35 | New |
| P17 | M | Pakistani | F8 | Homozygous | c.323_332del | p.F108YfsX11 | 39 | 39 | New |
| P18 | F | Pakistani | F8 | Homozygous | c.323_332del | p.F108YfsX11 | Asymptomatic | 3 | New |
| P19 | M | Caucasian | F9 | Compound heterozygous | c.58+1G>A  c.356T>C | -  p.F119S | 10 | 24 | New |
| P20 | F | Caucasian | F9 | Compound heterozygous | c.58+1G>A  c.356T>C | -  p.F119S | 1.5 | 23 | New |
| P21 | F | Iranian | F10 | Homozygous | c.451 C>G | p.V151L | 3 | 33 | New |
| P22 | M | Iranian | F10 | Homozygous | c.451 C>G | p.V151L | 6 | 35 | New |

**Table E4**. Clinical and genetic characteristics of 15 reported patients with ICOS deficiency

|  | **Previously reported patients** | | | | | | | | | | | | | | | |
| --- | --- | --- | --- | --- | --- | --- | --- | --- | --- | --- | --- | --- | --- | --- | --- | --- |
| **Family [ref]** | **Kindred A** [**^12^**](#_ENREF_12)**^,^** [**^14^**](#_ENREF_14)**^,^** [**^18^**](#_ENREF_18)**^,^** [**^33^**](#_ENREF_33) | | **Kindred B** [**^12^**](#_ENREF_12)**^,^** [**^14^**](#_ENREF_14)**^,^** [**^18^**](#_ENREF_18)**^,^** [**^33^**](#_ENREF_33) | | **Kindred C** [**^13^**](#_ENREF_13)**^,^** [**^14^**](#_ENREF_14)**^,^** [**^18^**](#_ENREF_18) | | **Kindred D** [**^13^**](#_ENREF_13)**^,^** [**^14^**](#_ENREF_14)**^,^** [**^18^**](#_ENREF_18) | | | **Kindred E** [**^15^**](#_ENREF_15)**^,^** [**^18^**](#_ENREF_18) | | **Kindred F** [**^17^**](#_ENREF_17)**^,^** [**^18^**](#_ENREF_18) | | **Kindred G** [**^16^**](#_ENREF_16)**^,^** [**^18^**](#_ENREF_18) | |  |
|  | **P1** | **P2** | **P3** | **P4** | **P5** | **P6** | **P7** | **P8** | **P9** | **P10** | **P11** | **P12** | **P13** | **P14** | **P15** |  |
| **Mutation** | g.18169  1,815 bp deletion | g.18169  1,815 bp deletion | g.18169  1,815 bp deletion | g.18169  1,815 bp deletion | g.18169  1,815 bp deletion | g.18169  1,815 bp deletion | g.18169  1,815 bp deletion | g.18169  1,815 bp deletion | g.18169  1,815 bp deletion | c.285 delT | c.285 delT | c.90 delG | c.90 delG | c.321_330 del | c.321_330 del |  |
| **Protein variation** | p.T19fsX9 | p.T19fsX9 | p.T19fsX9 | p.T19fsX9 | p.T19fsX9 | p.T19fsX9 | p.T19fsX9 | p.T19fsX9 | p.T19fsX9 | p.F95fsX26 | p.F95fsX26 | p.M30IfsX26 | p.M30IfsX26 | p.F108Y fsX10 | p.F108Y fsX10 |  |
| **Exon(s) affected** | Intron 1-intron 3 | Intron 1-intron 3 | Intron 1-intron 3 | Intron 1-intron 3 | Intron 1-intron 3 | Intron 1-intron 3 | Intron 1-intron 3 | Intron 1-intron 3 | Intron 1-intron 3 | Exon 2 | Exon 2 | Exon 2 | Exon 2 | Exon 2 | Exon 2 |  |
| **Ethnic Origin** | Caucasian  Danube region | Caucasian  Danube region | Caucasian  Danube region | Caucasian  Danube region | Caucasian  Danube region | Caucasian  Danube region | Caucasian  Danube region | Caucasian  Danube region | Caucasian  Danube region | Japanese | Japanese | Kuwaiti | Kuwaiti | Pakistani | Pakistani |  |
| **Consanguinity** | No | No | No | No | No | No | No | No | No | Yes | Yes | Yes | Yes | Yes | Yes |  |
| **Sex** | Female | Male | Male | Male | Male | Male | Female | Female | Male | Female | Male | Male | Female | Female | Male |  |
| **Presenting age, yr** | 27 | 19 | 6 | 12 | 20 | 12 | 8 | Asymptomatic | 1.5 | Infancy | 35 | One month | 3 | 2 | 2 |  |
| **Age at diagnosis, yr** | 28 | 21 | 31 | 22 | 38 | 41 | 15 | 4 | 1.5 | 34 | 35 | 6 months | 3 | 3.5 | 2 |  |
|  |  |  |  |  |  |  |  |  |  |  |  |  |  |  |  |  |
| **Hypogammaglobulinemia** | Yes | Yes | Yes | Yes | Yes | Yes | Yes | Yes | Yes | Yes | Yes  (Hyper IgM phenotype) | Yes  (Hyper IgM phenotype) | Mild (Transient HGG + SAD) | Yes | Yes |  |
| **Infections** | HPV, *Staphylococcus aureus* | HSV, *Giardia lamblia*, *Salmonella typhi.* *Campylobacter jejuni* |  | *Salmonella typhi* | HSV, *Borrelia burgdorferi* | HSV, HPV | HSV,CMV |  | HSV,CMV | CMV, HSV, *Helicobacter pylori*, *Escherichia coli* |  | Candida albicans, CMV, *Pneumocystis jerovecii* |  | HHV6  Norovirus, Adenovirus, Cryptosporidium |  |  |
| **Respiratory features** | URTI, LRTI | URTI, LRTI | URTI, LRTI, OME, BE | URTI, LRTI | URTI, LRTI, emphysema | URTI | Recurrent pneumonia, BE |  |  | Pulmonary abscess, interstitial pneumonitis |  | Candida-induced respiratory failure, PJP |  | URTI, LRTI |  |  |
| **GIT features** |  | Lambliasis, Salmonellosis. Campylobacter enteritis, NLH, SM |  | Recurrent salmonella enteritis, idiopatic hepatitis, HSM | SM, LN | HSM | CMV induced IBD |  | Pathogen-negative diarrhea, NLH, CMV colitis | IBD, CMV colitis |  | CD (chronic colitis, villous atrophy, crypt hyperplasia) | CD | CD HM, chronic hepatitis, high LFT, colitis | CD, high LFT, Idiopathic hepatitis |  |
| **Dermatologic features** | Impetigo | Eczema |  |  | Recurring localized herpes simplex, eczema | Recurring localized herpes simplex, verrucosis | Psoriasis, genital herpes |  | Psoriasis | Psoriasis-like lesions, CMV vulvovaginitis | Skin abscesses, psoriasis-like lesions |  |  | + toxic epidermal necrolysis |  |  |
| **Other features & complications** | Carcinoma of the vulva associated with HPV  Infection, giant granulomatous lesion in LN, alcoholic steatohepatitis | Recurrent herpes keratitis, NLH |  | Granulomatous, | CNS borreliosis, LN, IVIG reaction, lymphocytic meningoencephalitis | IgG-mediated autoimmune neutropenia, large granular lymphocyte T-cells with clonal expansion, pre-auricular squamous cell carcinoma grade I, persistent agranulocytosis |  |  | NLH, sterile arthritis | Prolonged viral infection in infancy, RA, Septic shock | Arthritis | CMV viremia |  | Fever, weight loss |  |  |
|  |  |  |  |  |  |  |  |  |  |  |  |  |  |  |  |  |
| **Treatment** | IVIG to SCIG  ANB | IVIG to SCIG, oral prednisolone, valacyclovir, keratoplasty | IVIG to SCIG,  ANB | IVIG to SCIG | IVIG to SCIG, ANB | SCIG, steroids, cyclosporine  HSCT | Lobectomy, Prophylactic ANB, steroids, intravenous acyclovir, oral valacyclovir, AM, IVIG | SCIG | IVIG to SCIG, steroids, intravenous acyclovir, oral valganciclovir | Prednisolone MTX, mesalazine, IVIG | steroids, ANB | TPN, IVIG, HSCT (Myeloablative) |  | Ganciclovir, valganciclovir, nitazoxanide, HSCT |  |  |
| **Outcome** | Dead  (44y, Carcinoma ) | Alive  (46y) | Alive  (47y) | Dead  (37y, Accident) | Alive  (56y) | Alive  (52y) | Alive  (28y) | Alive  (15y) | Alive  (15y) | Alive  (47y) | Alive  (44y) | Alive  (4y) | Alive  (10y) | Dead  (5y, capillary leak syndrome + toxic epidermal necrolysis) | Alive  (7yr) |  |

*ANB: antibiotics, AM: anti mycotic drugs, BE, bronchiectasis; CD, chronic diarrhea; CMV, cytomegalovirus; GIT, gastrointestinal; HM, hepatomegaly; HPV, human papillomavirus; HSCT, Hematopoietic stem cell transplantation; HSM, hepatosplenomegaly; IBD, inflammatory bowel disease; IVIG: intravenous immunoglobulin, LFT, liver function tests; LRTI, lower respiratory tract infections; LN: lymph node, NLH, nodular lymphoid hyperplasia; OM, otitis media; PJP, Pneumocystis jerovecii pneumonia; RA, rheumatoid arthritis; SCIG: subcutaneous immunoglobulin, SM, splenomegaly; TPN, total parenteral nutrition; URTI, upper*

*respiratory tract infections;*

**Table E5.** Clinical and genetic characteristics of 7 new patients with ICOS deficiency

|  | **New reported patients** | | | | | | |
| --- | --- | --- | --- | --- | --- | --- | --- |
| **Family [ref]** | **Kindred H** | **Kindred I** | **Kindred J** | **Kindred K** | | **Kindred L** | |
|  | **P16** | **P17** | **P18** | **P19** | **P20** | **P21** | **P22** |
| **Mutation** | c.323_332del | c.323_332del | c.323_332del | c.58+1G>A, c.356T>C | c.58+1G>A, c.356T>C | c.451 C>G | c.451 C>G |
| **Protein variation** | p.F108TfsX11 | p.F108TfsX11 | p.F108TfsX11 | Splicing mutation, p.F119S | Splicing mutation, p.F119S | p.V151L | p.V151L |
| **Exon(s) affected** | Exon 2 | Exon 2 | Exon 2 | Exon 2 | Exon 2 | Exon 3 | Exon 3 |
| **Ethnic Origin** | Pakistani | Pakistani | Pakistani | Caucasian | Caucasian | Iranian | Iranian |
| **Consanguinity** | No | Yes | Yes | No | No | Yes | Yes |
| **Sex** | Male | Male | Female | Male | Female | Female | Male |
| **Presenting age, yr** | 27 | 39 | Asymptomatic | 10 | 1.5 | 3 | 6 |
| **Age at diagnosis, yr** | 35 | 39 | 3 | 24 | 35 | 33 | 35 |
|  |  |  |  |  |  |  |  |
| **Hypogammaglobulinemia** | Yes | Yes | Yes | Yes | Yea | Yes | Yes |
| **Infections** | HPV, *Molluscum contagiosum* | TB, *Giardia lamblia*, Candida albicans, *Salmonella typhi* |  |  | HPV |  |  |
| **Respiratory features** | URTI,LRTI | URTI,LRTI, sinusitis, bronchial wall thickening | URTI/LRTI | LRTI | LRTI, bronchiectasis | Recurrent sinusitis, OME, pneumonia | Recurrent sinusitis, OME, |
| **GIT features** | SM, lymphocytic inflammatory liver disease, intermittent diarrhea, early sclerosing cholangitis | SM, lymphocytic inflammatory liver disease, severe lobar hepatitis, portal fibrosis and cholestasis |  | Coeliac disease | Inflammatory bowel disease | Chronic enteritis |  |
| **Other features & complications** | Delayed hypersensitivity to multiple antibiotics, granulomatous bone marrow infiltration (progressive pancytopenia), generalized lymphadenopathy | Lymphadenopathy |  |  | Juvenile rheumatoid arthritis, psoriasis, Delayed hypersensitivity to multiple immunoglobulin products |  |  |
|  |  |  |  |  |  |  |  |
| **Treatment** | IVIG, bleomycin, AM, ANB, ursodeoxycholic acid, prednisolone,  ustekinumab | IVIG, ursodeoxycholic acid, prednisolone | IVIG  hematopoietic stem cell transplantation | IVIG | Antibiotics  Prednisilone, Mesalazine, gold, | IVIG, antibiotics | Antibiotics |
| **Outcome** | Alive (47y) | Dead (53y, cirrhosis, pulmonary edema and salmonella sepsis) | Alive (4.5y) | Alive (32y) | Alive (36) | Alive (39y) | Alive (37y) |

*AM: anti mycotic drugs, ANB: antibiotics,* *IVIG: intravenous immunoglobulin, LRTI, lower respiratory tract infections; OME, otitis media; SM, splenomegaly; URTI, upper respiratory tract infections; TB, Tuberculosis.*

***Table E6.*** Immunological characteristics of 15 reported patients with ICOS deficiency

| **Previously reported patients** | | | | | | | | | | | | | | | |
| --- | --- | --- | --- | --- | --- | --- | --- | --- | --- | --- | --- | --- | --- | --- | --- |
| **Family** | **Kindred A** | | **Kindred B** | | **Kindred C** | | **Kindred D** | | | **Kindred E** | | **Kindred F** | | **Kindred G** | |
|  | **P1** | **P2** | **P3** | **P4** | **P5** | **P6** | **P7** | **P8** | **P9** | **P10** | **P11** | **P12** | **P13** | **P14** | **P15** |
| **Mutation** | p.T19fsX9 | p.T19fsX9 | p.T19fsX9 | p.T19fsX9 | p.T19fsX9 | p.T19fsX9 | p.T19fsX9 | p.T19fsX9 | p.T19fsX9 | p.F95fsX26 | p.F95fsX26 | p.M30IfsX26 | p.M30IfsX26 | p.F108Y fsX10 | p.F108Y fsX10 |
| **Age of evaluation, yr** | 29-42 | 21-43 | 35-46 | 25-34 | 46-53 | 42-52 | 15-28 | 4-15 | 3-15 | 34-48(BHSCT) | 35-48 | 0.5-3 | 10 | 3.5-6 | 2-8 |
| **Lymphocytes, per ul** | 1266 | 1047-800↓ | 1171-600↓ | 353↓ | 871↓ | 1722-270↓ | 3031 | 3584 | 4153 | 1400 | 1900 | NR | NR | 2900 | 5300 |
| **CD3+ cells, per ul** | 1210 | 926 | 908 | 326↓ | 629↓ | 816-250↓ | 2683 | 2727 | 2923 | 1127 | 1375 | 2514-300↓ | NR | 2352 | 2856 |
| **CD4+ Tcells, per ul** | 749 | 417↓ | 591 | 159↓ | 347↓ | 188↓ | 1095-500↓ | 1064 | 959 | 670 | 764 | 1394-100↓ | NR | 1748 | 1877 |
| **CD8 Tcells, per ul** | 403 | 494 | 266↓ | 119↓ | 104↓ | 522-140↓ | 1274 | 941 | 1017 | 271↓ | 262↓ | 1060-100↓ | NR | 469 | 879 |
| **CD4/CD8 ratio** | 1.9 | 0.8 | 2.2 | 1.3 | 3.3 | 0.4 | 0.9 | 1.1 | 0.9 | 2.5 | 2.9 | 1.3 | NR | 3.7 | 2.1 |
| **CD19 cells, per ul** | 8↓ | 13↓ | 32↓ | 11↓ | 127 | 33↓ | 184 | 686 | 882 | 23↓ | 66↓ | 477 | NR | 1607 | 2824 |
| **CD16 cells, per ul** | 40↓ | 50↓ | 200 | 30↓ | 30↓ | 7↓ | 147 | 163 | 80 | 190 | 30↓ | 76↓ | NR | 190 | 60↓ |
| **CD19 + CD27-IgD+ (% naïve B Cell)** | 89.6↑ | 88.5↑ | 94.9↑ | 92.9↑ | 90.4↑ | 90.2↑ | 96.8↑ | 95.2↑ | 97.7↑ | NR | NR | 98.8↑ | NT | 86 | 97↑ |
| **CD19 + CD27 + IgD+ (% memory B cell)** | 5.9↓ | 7.8↓ | 4.1↓ | 5.1↓ | 9 | 11.3 | 2.7↓ | 3.2↓ | 2↓ | 0.2↓ | 0.4↓ | 0.08↓ | NT | 2↓ | 3 ↓ |
| **CD19 + CD27 + IgD- (% class switched B Cell)** | 0.8↓ | 1.6↓ | 0.2↓ | 0.3↓ | 0.2↓ | 1.3↓ | 0↓ | 1.3↓ | 0↓ | 0↓ | 0.4↓ | 0.2↓ | NT | <1↓ | 0 ↓ |
| **CD19 + CD21 low CD38 low (% CD21low B cells )** | NT | NT | NT | NT | NT | NT | NT | NT | NT | NT | NT | 0.8↓ | NT | NR | NR |
| **CD4-CD45RA + CD27- (% effector CD8+)** | NT | NT | NT | NT | NT | NT | NT | NT | NT | NT | NT | NT | NT | 0 ↓ | 10 |
| **CD4-CD45RA + CD27+ (% naïve CD8+)** | NT | NT | NT | NT | NT | NT | NT | NT | NT | NT | NT | NT | NT | 75.2↑ | 84.4↑ |
| **CD4 + CD45RA + CD27+ (% naïve CD4+)** | 51.2 | 37.2 | 78.2↑ | 30.7 | 60.4 | 44.5 | 56.2 | 87.3↑ | 81.8↑ | High | High | NT | NT | 40.3 | 42.5 |
| **Activated T cells (% HLA-DR+)** | Normal | Normal | Normal | Normal | NR | NR | NR | NR | NR | Normal | Normal | NT | NT | 5 | 7 |
| **CD4+ CD25+ FOXP3+ T cells (% T cell)** | NT | NT | NT | NT | NT | NT | NT | NT | NT | Normal | Normal | 3.86% | NT | NT | NT |
| **CD45RO+ CD4+ helper T cells (% T cell)** | 39.5 | 50.6 | 23.2↓ | 60.0 | 38.1 | 48.2 | 34.5 | 14.8↓ | 12.9↓ | 12.1↓ | 6.6↓ | NT | NT | NT | NT |
| **CD45RA- CD45RO+ CXCR5hi CD4+ follicular helper T cells (% T cell)** | <5↓ | <5↓ | <5↓ | <5↓ | <5↓ | <5↓ | <5↓ | <5↓ | <5↓ | NR | NR | NT | 1.85↓ | NT | 2.9 ↓ |
| **SHM % bp** | NR | 3.8 | NT | NR | 2.2↓ | ND | NT | 2.3↓ | NT | NT | NT | NT | NT | NT | NT |
| **LTT, proliferation in vitro response** | Normal | Normal | Normal | Normal | NT | NT | NT | NT | NT | Normal | Normal | Normal | Normal | NT | NT |
| **IgM, mg/dl** | <20↓ | <20↓ | 26↓ | 31↓ | 38↓ | 180 | 26↓ | 57 | 43 | 56 | 456↑ | 203 | Normal | 17 ↓ | 32↓ |
| **IgG, mg/dl** | <190↓ | 404↓ | 249↓ | 105↓  Normal G3 | 101↓  Normal G3 | 57↓ | 255↓  Normal G3 | 213↓ | 54↓ | 315↓ | 611↓ | 113↓ | 654 | 207↓ | 150↓ |
| **IgA, mg/dl** | <30↓ | 40↓ | 46↓ | <6↓ | <6↓ | <6↓ | 25↓ | 58↓ | 20↓ | 46↓ | 103 | 3/0↓ | Normal | 43↓ | 29↓ |
| **IgE, IU/ml** | <28 | <17.5 | <17.5 | <17.5 | <17.5 | <19.1 | <30 | NR | <17.5 | <5 | <5 | 1 | NR | NT | NT |
| **Anti-Tetanus, IU/ml** | NT | NT | Low | Low | NT | NT | NT | Low | Low | NT | NT | NT | protective | non-protective | non-protective |
| **Anti-Diphtheria, IU/ml** | NT | NT | Low | Low | NT | NT | NT | Low | low | NT | NT | NT | NT | NT | NT |
| **Anti-Hepatitis A** | NT | NT | Low | Low | NT | NT | NT | NT | NT | NT | NT | NT | NT | NT | NT |
| **Anti-Hib,** **ųg/ml** | NT | NT | NT | NT | NT | NT | NT | NT | NT | NT | NT | NT | NT | 0.36 | 0.02 |
| **Pneumococcal serotypes: protective responses** | NT | NT | NT | NT | NT | NT | NT | NT | NT | NT | NT | NT | 3/23 serotypes | 0/12 serotypes | 2/12 serotypes |
| **Measles IgG** | NT | NT | NT | NT | NT | NT | NT | NT | NT | Low | Low | NT | NT | NT | Positive |
| **Mumps IgG** | NT | NT | NT | NT | NT | NT | NT | NT | NT | Low | Low | NT | NT | NT | Positive |
| **Rubella IgG** | NT | NT | NT | NT | NT | NT | NT | NT | NT | Low | Low | NT | NT | NT | Negative |
| **Neoantigen Phage Φ174** | Low | Low | NT | NT | NT | NT | NT | NT | NT | NT | NT | NT | NT | NT | NT |
| **Other Investigation** | Loss of ICOS protein, Normal CD28 response, Low IL10, IL17 | Loss of ICOS protein, Normal CD28 response, Low IL10, IL17 | Loss of ICOS protein, Relative increase pre-B1 B cells in BM | Loss of ICOS protein, Relative increase pre-B1 B cells in BM | Relative increase pre-B1 B cells in BM | Low activated T cells | Low activated T cells | Low activated T cells | Low activated T cells | Low IL-10+ICOS+ CTLA4+ Tregs, reduced CTLA4+ activated T cells, low Th17 | Low IL-10+ICOS+ CTLA4+ Tregs, reduced CTLA4+CD4+ and PD1+CD8+ activated T cells, low Th17 | Loss of ICOS protein, Increased CD25 after PHA stimulation | Loss of ICOS protein, Increased CD25 after PHA stimulation | Low activated T cells | Loss of ICOS protein, Increased CD69 after PHA stimulation |

***Table E7*.** Immunological characteristics of 7 new patients with ICOS deficiency

|  | **New reported patients** | | | | | | |
| --- | --- | --- | --- | --- | --- | --- | --- |
| **Family** | **Kindred H** | **Kindred I** | **Kindred J** | **Kindred K** | | **Kindred L** | |
|  | **P16** | **P17** | **P18** | **P19** | **P20** | **P20** | **P21** |
| **Mutation(s)** | p.F108TfsX11 | p.F108TfsX11 | p.F108TfsX11 | Splice site + p.F119S | Splice site + p.F119S | p.V151L | p.V151L |
| **Age of evaluation, yr** | 44 | 39 | 3 | 24 | 23 | 33 | 37 |
| **31Lymphocytes, per ul** | 1320 | 1980 | 9802 | 2370 | 4142 | 1803 | 2430 |
| **CD3+ cells, per ul** | 1156 | 1584 | 5581 | 1810 | 3490 | 1357 | 1580 |
| **CD4+ Tcells, per ul** | 651 | 871 | 3786 | 1257 | 2295 | 724 | 610 |
| **CD8 Tcells, per ul** | 494 | 673 | 1650 | 582 | 1189 | 652 | 932 |
| **CD4/CD8 ratio** | 1.32 | 1.29 | 2.29 | 2.16 | 1.93 | 1.1 | 0.6 |
| **CD19 cells, per ul** | 125 | 40↓ | 3849 | 346 | 421 | 81 | 253 |
| **CD16 cells, per ul** | 89↓ | NT | NT | NT | NT | 142 | 180 |
| **CD19 + CD27-IgD+ (% naïve B Cell)** | 95↑ | NT | NT | 90↑ | 67 | 95.3↑ | 92↑ |
| **CD19 + CD27 + IgD+ (% memory B cell)** | 5 | NT | NT | 4↓ | 25 | 1.5↓ | 2↓ |
| **CD19 + CD27 + IgD- (% class switched B Cell)** | 0 | NT | NT | 1↓ | 3.36 ↓ | 0.4↓ | 0.8↓ |
| **CD19 + CD21 low CD38 low (% CD21low B cells )** | NT | NT | NT | NT | 10 ↑ | 1↓ | 1.5↓ |
| **CD4+ CD25+ FOXP3+ (% T cell)** | Reduced | NT | NT | NT | NT | 5.7 | 6.5 |
| **LTT, proliferation in vitro response** | Reduced | Reduced | NT | Normal | Reduced | Normal | Normal |
| **IgM, mg/dl** | 20↓ | 24↓ | 13↓ | 37↓ | 150 normal | 10↓ | 26↓ |
| **IgG, mg/dl** | 310↓ | 270↓ | <10↓ | 170↓ | 260 low | 100↓ | 525↓ |
| **IgA, mg/dl** | 11↓ | 44↓ | 23↓ | 57↓ | 100 normal | 0↓ | 6↓ |
| **IgE, IU/ml** | NT | NT | NT | NT9 | NT | 0 | <3 |
| **Anti-Tetanus, IU/ml** | 0.04 | 0.011 | <0.01 | 0.02 | 0.02 | 1.3 | 1.8 |
| **Anti-Diphtheria, IU/ml** | NT | NT | NT | NT | NT | 0.1 | 0.1 |
| **Anti-Hepatitis A** | NT | NT | NT | NT | NT | NT | NT |
| **Anti-Hib,** **ųg/ml** | 0.71 | 0.13 | <0.1 | 2.84 | 1.24 | 0.17 | 0.88 |
| **Pneumococcal serotypes: protective responses** | NT | 1.26↓ | <3.3 | 6.7 | 6 mg/L | 2/23 serotypes | 3/23 serotypes |
| **Other Investigation** | Loss of ICOS protein | Loss of ICOS protein | NT | Loss of ICOS protein | Loss of ICOS protein | NT | NT |
